# A linear models approach to optimize carbazole-based dyes for solar cell applications

**DOI:** 10.1007/s10822-026-00874-7

**Published:** 2026-07-06

**Authors:** Emanuel F. dos S. Mattos, Carlos R. A. Daniel, Nivan B. da Costa Júnior

**Affiliations:** 1 Department of Chemistry, Federal University of Sergipe Foundation, Sergipe, 49100-000 Brazil; 2 Department of Statistics, Federal University of Sergipe Foundation, Sergipe, 49100-000 Brazil

**Keywords:** Solar energy, MLR, Metal-free, Quantitative structure-activity relationship, DFT

## Abstract

The excellent characteristics of carbazole-based dyes have resulted in DSSCs with power conversion efficiencies (PCEs) that are among the highest for metal-free dyes. However, the rational modification of these structures remains a challenge. In this work, 126 carbazole-based dyes were used in the development of models to predict the efficiency of DSSCs and to describe the relation between dye structure and cell efficiency. Using variable selection techniques (genetic algorithm and best subsets) on a pool of 2393 molecular descriptors (2D, 3D, quantum, and spectroscopic), three multiple linear regression models were obtained that satisfied well-established validation criteria (e.g., R^2^_Pred_ > 0.7, Q^2^_LOO_ > 0.6, r^2^_m (LOO)_ > 0.5 and, ∆r^2^_m (LOO)_ < 0.2). The best model indicated important molecular characteristics: π-bridge length, increased molecular branching, ionization potential, and presence of electronegative atoms. Based on these features, 36 novel sensitizers were designed, three of which achieved a predicted PCE approaching 9%, representing a potential improvement of almost two times over the reference sensitizers (LY‑S, LY‑P, and LY‑F). When we evaluated a small sample of the modified structures using common DFT and TD-DFT calculations for assessing new sensitizers, a favorable tuning of the electronic and spectroscopic properties was observed, which corroborates the predictions made by the models. Therefore, the best model presented can be employed for the rapid screening of carbazole-based sensitizers.

**Supplementary Information:**

The online version contains supplementary material available at 10.1007/s10822-026-00874-7.

## Introduction

 Global energy demands are predominantly met by fossil fuels, resulting in significant geopolitical and environmental challenges [1, 2]. Given the projected increase in energy consumption over the coming decades, these issues are anticipated to intensify further [3]. In this context, solar energy has emerged as a promising alternative, owing to advancements in photovoltaic technology that have substantially improved its cost-effectiveness [4, 5]. Among various solar cell technologies, dye-sensitized solar cells (DSSCs) stand out due to their facile synthesis, low production costs, and high light-to-electricity conversion efficiency under diverse lighting conditions [6].

Since their discovery [7], DSSCs have retained a fundamental structure comprising a photoanode, a cathode, and an electrolyte system. The photoanode consists of a semiconductor oxide film (e.g., TiO_2_ or ZnO) deposited on a conductive transparent substrate (typically fluorine-doped tin oxide, FTO). The electrolyte system, which facilitates charge transport, usually contains a redox mediator (e.g., iodide/triiodide or cobalt-based complexes) dissolved in an organic solvent [8, 9]. The energy conversion mechanism involves multiple charge-transfer steps: (i) upon illumination, the dye molecule absorbs photons, promoting it to an excited state; (ii) the excited dye injects electrons into the conduction band of the semiconductor; (iii) these electrons diffuse through the semiconductor network toward the anode, generating electrical current; and (iv) the oxidized dye is regenerated by the redox mediator, completing the circuit. Since the sensitizer initiates this process, its photophysical and electrochemical properties critically determine the overall cell efficiency [9–11]. Thus, the molecular engineering of high-performance sensitizers remains a crucial research direction for enhancing DSSC efficiency.

Among various sensitizer dyes, metal-free organic variants have attracted attention due to their eco-friendly nature and structural diversity [12–14]. These dyes typically feature three key components: (i) a donor (D) group, for light absorption; (ii) a π-conjugated bridge (π) that enables charge separation; and (iii) an acceptor (A) group that anchors the dye to the semiconductor and accept the excited electrons. Diverse combinations of these groups give rise to distinct molecular architectures, including D-A-π-A [15], D-D-π-A [16], (D-A)_2_ [17], D-π-A-π-A [18, 19], and A-π D-π-A [13], among which the D-π-A has been most extensively explored in literature [20–22]. Among various donor groups, carbazole-based donors have emerged as particularly promising materials due to their synthetic accessibility, structural stability, and multifunctional properties. Beyond photovoltaics, carbazole derivatives find extensive applications in medicinal chemistry [23, 24] and luminescent materials [25, 26], owing to their facile synthesis, straightforward purification, and ease of functionalization [27–30]. These compounds exhibit exceptional chemical, thermal, and photochemical stability [31–33], coupled with efficient hole-transport properties [34] and strong light-harvesting capabilities [35], making them ideal candidates for high-efficiency DSSCs [36, 37].

While carbazole-based dyes have shown considerable promise in DSSCs, only a limited number of studies report power conversion efficiencies (PCEs) approaching or exceeding 10%. Soni et al. [38] achieved a PCE of 9% through electrolyte optimization, demonstrating that replacing the conventional iodide/triiodide redox mediator with a cobalt-based (Co^2+^/Co^3+^) system enhanced device performance by approximately 30%. Furthermore, Liu et al. [37] reported a novel π-bridge design incorporating a cyclic structure, which enabled the DSSC to achieve an impressive PCE of 9.20%. However, this system’s practical implementation is limited by its multi-step synthesis and low overall reaction yield. The most notable performance was reported by Kakiage et al. [36, 39] with their ADEKA-1 dye, featuring a trimethoxysilyl anchoring group, which achieved a remarkable PCE exceeding 12%. However, like the system by Liu et al. [37], the synthesis of the ADEKA-1 is complex and yields are suboptimal, posing challenges for practical applications.

Overall, the rational design and cost-effective development of high-efficiency carbazole-based dyes remains a significant challenge in DSSC research. In this context, quantitative structure-property relationship (QSPR) modeling offers a promising approach to elucidate structure-performance correlations in DSSCs, enabling the rational design of novel high-efficiency carbazole-based sensitizers. This method establishes mathematical relationships between molecular structures (described by molecular descriptors, MDs) and target properties such as power conversion efficiency (PCE). By identifying and interpreting significant MDs within these models, researchers can strategically optimize dye structures to enhance DSSC performance. Thus, this study employs linear regression modeling to develop robust predictive tools specifically for the power conversion efficiency (PCE) of carbazole-based organic dyes in DSSCs. Furthermore, the molecular descriptors (MDs) in the optimal model were analyzed to extract chemical insights, guiding more effective structural modifications. Building on these insights, we strategically modify π-bridge architectures in a series of reference carbazole dyes, demonstrating theoretically enhanced photovoltaic performance.

## Methodology

### Dye structures and conversion efficiencies

The structures of the carbazole-based dyes and the experimental data for DSSCs were retrieved from an open-access database [40], which provides a diversity of experimentally validated dyes regarding their application as sensitizers in solar cells. Initially, the database contained about 600 carbazole-based dyes, but in order to attribute the variation in PCE to the sensitizer, a set of filters was applied. More precisely, only dyes/cells that possess the following were selected: carbazole as the donor group; TiO_2_ as the semiconductor oxide in the photoanode; platinum as the counter electrode; I^−^/I_3_^−^ as the redox pair; absence of a co-sensitizer; and cyanoacrylic acid as the only anchoring group of the sensitizer. Thus, the application of these criteria resulted in the identification of 126 cells, together with their corresponding sensitizers, whose architecture includes, for example, D-A-π-A, D-π-A-π-A and D-π-A.

## Structural optimization of dyes

The initial structures of the sensitizers were designed using ChemSketch 2021.1.0 and optimized in the gas phase. The optimization was performed using the semi-empirical RM1 method [41] from MOPAC2016 [42], with a convergence criterion of GNORM = 0.1. The POLAR keyword was employed to calculate the polarizability tensor.

## Obtaining molecular descriptors

After optimizing the dye geometries, 1875 structural descriptors (1444 in 2D and 431 in 3D) were calculated using PaDEL-Descriptor [43], while 18 descriptors were derived from quantum calculations. Absorptions in the visible region were also incorporated as molecular descriptors. The theoretical spectra were generated by computing the excited states of the optimized structures using MOPAC2016 with the INDO/S Hamiltonian [44], considering the solvent employed in the study reporting the dye. Theoretical spectra in the 400–600 nm region were then obtained by Lorentzian fitting, with a half-height bandwidth of 25 cm^−1^.

## Training and test group division

The principal component analysis (PCA) and hierarchical cluster analysis (HCA) techniques were used to analyze the data and understand how the dyes grouped together. For PCA, the data were rescaled and new dimensions were identified when the principal components explained 90% of the initial variance. Subsequently, the PCA score matrix was used to split the data into training and test groups. The HCA was performed using Euclidean distances and Ward’s method for similarity measurement and object agglomeration. Approximately 75% of each cluster was randomly assigned to the training set, while the remaining 25% was used for the test group. The training and test sets included 95 and 31 structures, respectively. All these calculations employed the FactoMineR [45] library in the RStudio [46] environment.

## Model development and validation

Firstly, the original matrix was reduced by removing variables with low variance (σ^2^ < 10^−6^) and high correlation (*r* > 0.95) between descriptors. Modeling was then performed by multiple linear regression (MLR), employing the genetic algorithm (GA) and best subsets methods for variable selection. Variable selection was performed in two steps. First, a genetic algorithm (GA) was used with the following parameters: 250 individuals; 500 generations; mutation probability of 0.05; single-point crossover; and a minimum and maximum of 5 and 12 variables, respectively. Then, models satisfying $\:{\mathrm{R}}_{\mathrm{T}\mathrm{r}\mathrm{a}\mathrm{i}\mathrm{n}}^{2}$ > 0.6, $\:{\mathrm{R}}_{\mathrm{P}\mathrm{r}\mathrm{e}\mathrm{d}}^{2}$ > 0.5, and, for each descriptor,* p*-value ≤ 0.05 and VIF ≤ 5 were selected. In the second step, the models with the highest $\:{\mathrm{R}}_{\mathrm{T}\mathrm{r}\mathrm{a}\mathrm{i}\mathrm{n}}^{2}$ and $\:{\mathrm{R}}_{\mathrm{P}\mathrm{r}\mathrm{e}\mathrm{d}}^{2}$ were chosen for a refinement process using the best subsets method. Here, a model was considered refined if it presented$\:\:{\mathrm{R}}_{\mathrm{T}\mathrm{r}\mathrm{a}\mathrm{i}\mathrm{n}}^{2}$ ≥ 0.7, $\:{\mathrm{R}}_{\mathrm{P}\mathrm{r}\mathrm{e}\mathrm{d}}^{2}$ ≥ 0.65, * p*-value r ≤ 0.05 and VIF ≤ 5 for each descriptor, and a maximum of 14 variables. Thus, the most predictive models were validated using metrics commonly reported in the literature, including coefficients of determination, $\:{\mathrm{R}}_{\mathrm{T}\mathrm{r}\mathrm{a}\mathrm{i}\mathrm{n}}^{2}$ and $\:{\mathrm{R}}_{Adjusted\left(Adj\right)}^{2}$, and leave-one-out cross-validation for predictive capacity evaluation.

## Results and discussion

### QSPR models and validations

In the first phase of variable selection, the genetic algorithm generated 198 models, which were then reduced to the top 15 most promising candidates for further refinement. The best subsets method, together with new selection criteria, was then used to find three models, described by Eqs. 1, 2, 3. These PCE models satisfied the following conditions used to indicate a satisfactory QSPR model [47]: (i) *n* > 4 k’, where n is the number of structures in the training set and *k’* is the number of descriptors in the model; (ii) all the descriptors are statistically significant (*p*-value < 0.05); (iii) all the descriptors have a correlation coefficient lower than a certain threshold (e.g., < 0.8).

Model 1 (M-1):

1 $$ \begin{aligned} PCE = & ~41.463 + 1.463~ATSC4e - 0.121~ATSC4s \\ & \quad - 5.876~GATS7c - ~98.970~VE2_{{Dzi}} \\ & \quad + 0.161~C1SP3 - 0.986~minHBint8~ \\ & \quad - 5.712~MDEO_{{11}} + 5.095 \times 10^{{ - 2}} ~PNSA_{3} \\ & \quad - 6.508~SpMax2_{{Bhp}} + 6.561 \times 10^{{ - 3}} ~COSMO_{{area}} \\ \end{aligned} $$

Model 2 (M-2):

2 $$ \begin{aligned} PCE = & ~39.827 - 0.3491~ALogP \\ & \quad + 1.412~ATSC4e - 0.122~ATSC4s \\ & \quad - 2.139~ATSC7c - 5.335~GATS7c \\ & \quad - 84.194~VE2_{{Dzi~}} - 1.213~minHBint8 \\ & \quad - 5.871~SpMax2_{{Bhp}} - 8.282~RotBFrac \\ & \quad + 0.230~C1SP3 - 11.458~MDEO_{{11}} \\ & \quad + 4.125 \times 10^{{ - 3}} ~COSMO_{{area}} - 5.396~BCUTc_{{1l}} \\ \end{aligned} $$

Model 3 (M-3):

3 $$ \begin{aligned} PCE = ~ & 37.65727 + 2.34 \times 10^{{ - 4}} ~ATS6m \\ & \quad + 2.015 \times 10^{{ - 2}} ATSC6i - 22.955~MATS4s \\ & \quad - 4.413~GATS7c + 4.710~AATSC5s \\ & \quad - 61.177~VE2_{{Dzv}} - 10.921~BCUTc_{{1l}} \\ & \quad - 12.646~SpMin1_{{Bhi}} + 4.687 \times 10^{{ - 2}} ~VE3_{{Dt}} \\ & \quad - 1.054~ndO + 5.691~FNSA_{2} - 1.017~minHBint8 \\ & \quad - 9.164 \times 10^{{ - 2}} ~SaasC + 0.839~I_{{434.46}} \\ \end{aligned} $$

The $\:{\mathrm{R}}_{\mathrm{T}\mathrm{r}\mathrm{a}\mathrm{i}\mathrm{n}}^{2}$ (Table 1) values showed that the models could effectively explain a significant portion of the data variability, with the M-3 exhibiting the highest value ($\:{\mathrm{R}}_{\mathrm{T}\mathrm{r}\mathrm{a}\mathrm{i}\mathrm{n}}^{2}=0.85$) among all models. Moreover, there was a strong correlation between $\:{\mathrm{R}}_{\mathrm{T}\mathrm{r}\mathrm{a}\mathrm{i}\mathrm{n}}^{2}$ and $\:{\mathrm{R}}_{\mathrm{A}\mathrm{d}\mathrm{j}}^{2}$. The $\:{Q}_{LOO}^{2}$ values higher than 0.6 indicated that there was no overfitting and that the models had good internal predictability [48, 49], which was supported by the similarity between the error measures for the fits (RMSE and MAE) and cross-validations (RMSE_CV_ and MAE_CV_). Thus, based solely on the training set metrics, the decreasing order of model performance is M-3 > M-2 > M-1.

**Table 1:** Variance explained, internal predictability, and error measures for the training and test sets. (bold text indicates the best metrics)

Model	Training							Test		
	$\:{\mathrm{R}}_{\mathrm{T}\mathrm{r}\mathrm{a}\mathrm{i}\mathrm{n}}^{2}$	$\:{\mathrm{R}}_{\mathrm{A}\mathrm{d}\mathrm{j}}^{2}$	$\:{\mathrm{Q}}_{\mathrm{L}\mathrm{O}\mathrm{O}}^{2}$	RMSE	RMSE_CV_	MAE	MAE_CV_	$\:{\mathrm{R}}_{\mathrm{P}\mathrm{r}\mathrm{e}\mathrm{d}}^{2}$	RMSE_Pred_	MAE_Pred_
M-1	0.734	0.702	0.630	0.986	1.162	0.771	0.891	0.709	0.858	0.694
M-2	0.812	0.781	0.711	0.829	1.026	0.668	0.804	0.709	0.859	0.709
M-3	**0.846**	**0.819**	**0.781**	**0.750**	**0.894**	**0.577**	**0.692**	**0.743**	**0.807**	**0.659**

It is commonly acknowledged that a low Q^2^ value indicates low predictive ability of a model. However, it is not correct to assume that a high Q^2^ value always reflects a strong ability to predict the properties of compounds not included in the training set [50]. Therefore, a test set and its metrics are used to evaluate a model’s predictive performance. Thus, among the various metrics employed to the test set, the $\:{R}_{Pred}^{2}$ values higher than 0.5 are widely considered indicative of a model’s strong predictive ability [51]. Overall, all the three models have a good predictive ability (Table 1), with an ascending order of predictivity M-1 ≈ M2 < M-3.

**Fig. 1:**
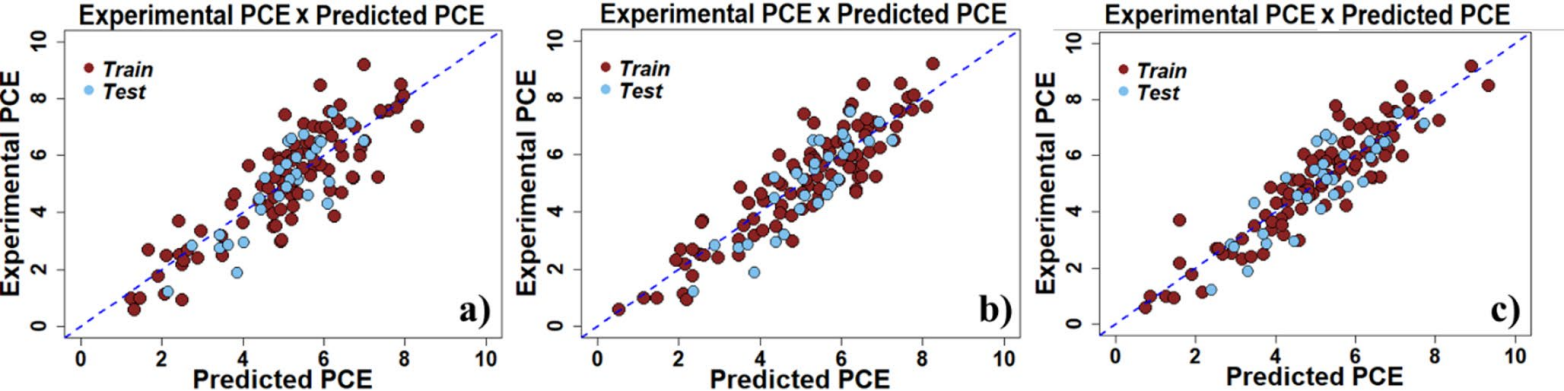
Scatter plots of the experimental PCE values against the predicted values for the training and test sets, for **a** M-1, **b** M-2, and **c** M-3

Furthermore, additional metrics—proposed by Golbraikh and Roy [48, 50] – were calculated as supplementary parameters for model evaluation (Table 2). As shown in Table 2, both training and test sets for all models achieved the required threshold values across all evaluated metrics: r^2^_m (LOO)_ > 0.5, $\Delta{r}$^2^_m(LOO)_ > 0.2, 0.85 ≤ k ≤ 1.15, and $\left[ {\left( {{\mathrm{r}}^{2} - {\mathrm{R}}_{{\mathrm{o}}}^{2} } \right)/{\mathrm{r}}^{2} } \right]$ < 0.1. Then, these results confirm the predictive order: M-1 ≈ M2 < M-3. Therefore, as shown in Tables 1 and 2, all the models satisfied the established QSPR validation criteria [48, 51, 52] and the analysis of the proposed models showed that M-1 had the poorest performance, while M-3 provided the strongest fits and the best predictive capacity. Visually, strong correlations between the predicted and experimental PCE values (Fig. 1A–C) confirmed the robust predictive capabilities of the models.

**Table 2:** Metrics for internal and external validation of the models developed. (bold text indicates the best metrics)

Model	Training		Test		
	$\:{r}_{m\:\left(LOO\right)}^{2}$	$\Delta r_{{m\left( {LOO} \right)}}^{2}$	$\:{r}_{m}^{2}$	$\:k$	$\:\frac{{r}^{2}-{R}_{o}^{2}}{{r}^{2}}$
M-1	0.506	0.189	0.614	1.003	0.037
M-2	0.607	0.151	0.611	0.964	0.053
M-3	0.696	0.126	**0.698**	**0.975**	**0.007**

**Fig. 2:**
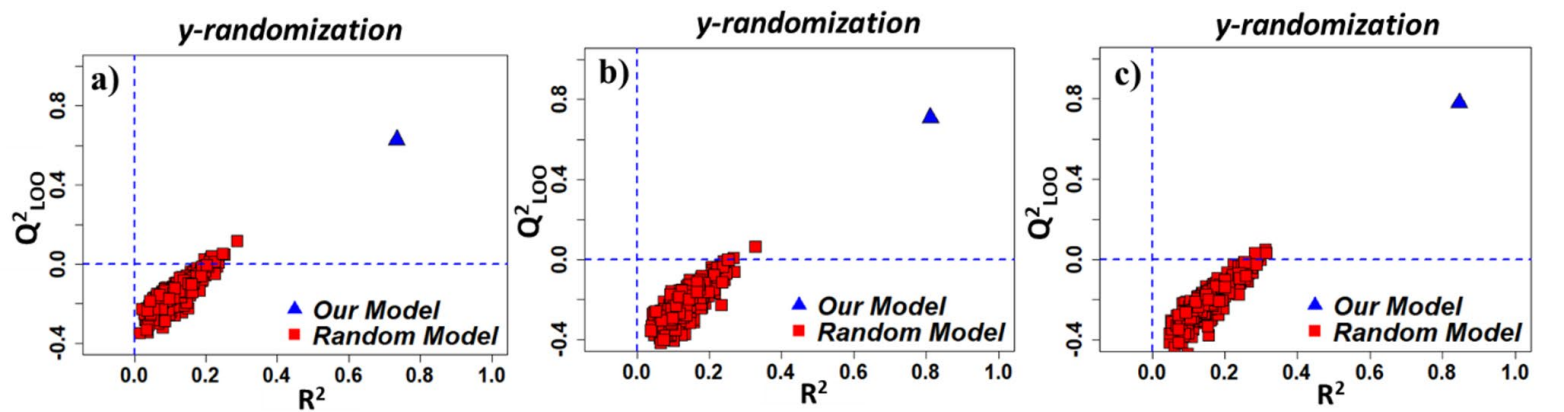
Plots of Q^2^ against R^2^ for the y-randomization tests applied to models **a** M-1, **b** M-2, and **c** M-3

The significance of the models was assessed by y-randomization (500 iterations), recording the R^2^ and Q^2^ values for each random model. Figure 2 shows a comparison of the performance of the developed models and random models, using Q^2^ and R^2^ metrics. The results showed that the developed models presented significantly higher coefficients of determination when compared to the random models, indicating the relevance of the selected descriptors for predicting PCE [53].

**Fig. 3:**
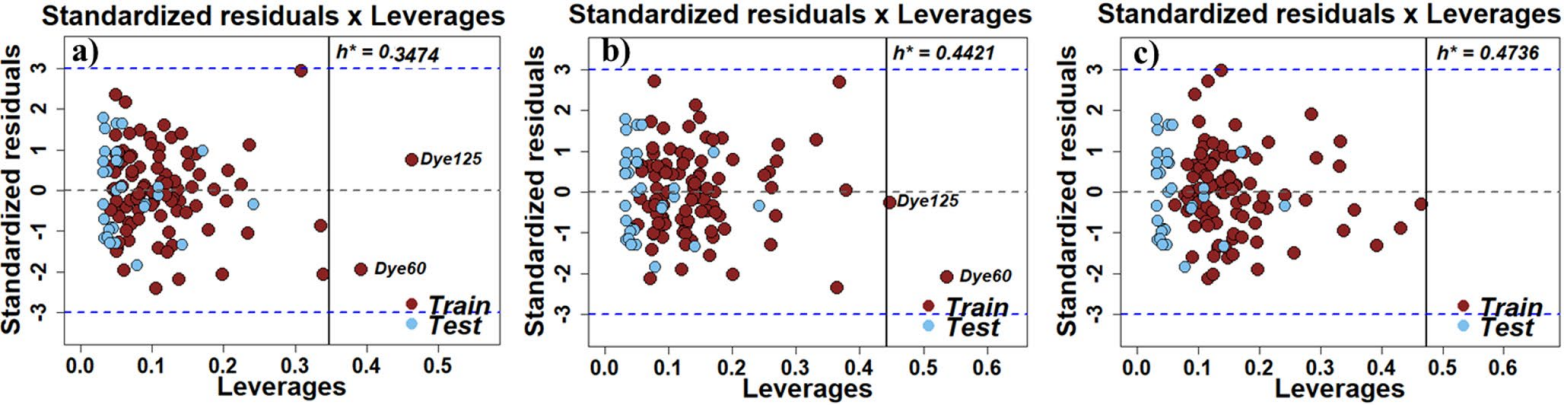
Willams plots for models **a** M-1, **b** M-2, and **c** M-3

In addition to assessing predictive ability, it is essential to establish the applicability domain (AD) of a QSPR model. Therefore, it was determined whether the studied compounds were within the AD of each model [54]. A common method uses leverage values, with leverage below h* indicating applicability (h^*^ *= 3p/n*, where *n* is the training set size and *p* represents model terms) [55–57]. The construction of Williams plots (Fig. 3) showed standardized residuals within ± 3 for all compounds in M-1, M-2, and M-3, indicating an absence of outliers. However, only M-3 showed leverage values below the threshold (h* = 0.4736) for all the compounds. In M-1 (h* = 0.3474) and M-2 (h* = 0.4421), the leverage values for Dye60 and Dye125 exceeded the threshold, suggesting unreliable property predictions for these dyes [58].

Thus, the linear models obtained for carbazole-based dyes demonstrate a similar performance to models that employ both more sophisticated regression algorithms and computationally more expensive molecular descriptors. For example, using molecular descriptors derived from DFT calculations and algorithms such as RF (Random Forest) and ANN (Artificial Neural Network), Yaping Wen et al. [59] obtained models for the PCE of DSSCs with a Pearson correlation coefficient for the test set of approximately 0.8. Another similar example was reported by Liao et al. [60], who employed convolutional neural networks to predict the efficiency of porphyrin-based DSSCs, achieving a test set performance similar to that of Yaping et al. [59]. In other words, the models reported here (M-1 to M-3), in addition to presenting performances similar to other DSSC models, are: (i) specific to carbazole-based dyes; (ii) establish direct relationships between molecular descriptors and the cell PCE; and (iii) the descriptors can be quickly obtained since only semi-empirical methods were employed. These points favor the rapid screening of potential new carbazole-based sensitizers.

### Interpretation of molecular descriptors

In some cases, an informative interpretation of the molecular descriptors in a model can be a challenging task. Therefore, to extract insights from the molecular descriptors, our approach involved correlating some descriptors with chemically interpretable molecular properties. More specifically, we focused our analysis only on model 3 (M-3), which demonstrated the most robust performance across both the training and the test sets.

Consequently, among the list of descriptors, $\:{\mathrm{I}}_{434.46}$ clearly indicates the need for dyes exhibiting strong absorptions in the visible region (434 nm) to enable solar cells with higher predicted PCEs. In this sense, this descriptor is associated with the light-harvesting efficiency (LHE) and the requirement for intense absorption in the visible region, which are characteristics strongly linked to a good sensitizer [61, 62]. However, beyond intense absorption, the DSSC efficiency is also directly impacted by the spectral overlap between the charge-transfer band—commonly associated with the maximum absorption (λ_max_)—and the solar radiation spectrum [62–64]. More precisely, a greater spectral overlap favors an increase in the number of electrons injected into the conduction band of the semiconductor, leading to a rise in PCE due to the increase in short-circuit current density, Jsc [65, 66]. Thus, both the descriptor $\:{\mathrm{I}}_{434.46}$ and the spectral overlap can be enhanced by increasing the π-conjugation of the sensitizer, leading to an increase in cell efficiency.

Additionally, other structural aspects are suggested by the descriptor $\:{\:\mathrm{F}\mathrm{N}\mathrm{S}\mathrm{A}}_{2}$(Fractional Negative Surface Area), as given by Eq. 4 – for further details, see [67]. $\:\mathrm{F}\mathrm{N}\mathrm{S}{\mathrm{A}}_{2}$ is a molecular descriptor that measures the exposure of negatively charged atomic sites to solvent molecules. Its definition is given by Eq. 4.

4 $$\:\mathrm{F}\mathrm{N}\mathrm{S}{\mathrm{A}}_{2}=\:\frac{{\mathrm{Q}}^{-}{\sum\:}_{{a}^{-}}^{}{\mathrm{S}\mathrm{A}}_{a}^{-}}{\mathrm{S}\mathrm{A}\mathrm{S}\mathrm{A}}$$

Where $\:{\mathrm{Q}}^{-}$ is the sum of all partial negative charges in the molecule, $\:{\mathrm{S}\mathrm{A}}_{\mathrm{a}}^{-}$ is the solvent-accessible surface area of a negatively charged atom $\:a$, and $\:\mathrm{S}\mathrm{A}\mathrm{S}\mathrm{A}$ corresponds to the total solvent-accessible surface area of the molecule. Increasing the $\:\mathrm{S}\mathrm{A}\mathrm{S}\mathrm{A}$ reduces the absolute value of $\:{\mathrm{F}\mathrm{N}\mathrm{S}\mathrm{A}}_{2}$, and this reduction is associated with a rise in the predicted PCE. One satisfactory strategy to enhance the $\:\mathrm{S}\mathrm{A}\mathrm{S}\mathrm{A}$ of the sensitizer is to modify its molecular structure. For example, elongating the π-bridge or adding substituents can increase molecular bulkiness. In this regard, the use of linear alkyl chains can provide a considerable increase in the $\:\mathrm{S}\mathrm{A}\mathrm{S}\mathrm{A}$ of the sensitizers, contributing to the reduction of the $\:{\mathrm{F}\mathrm{N}\mathrm{S}\mathrm{A}}_{2}$ descriptor. Experimentally, the insertion of alkyl chains is commonly linked to the reduction of undesirable π-π interactions, promoting an increase in PCE via an increase in Jsc and the open-circuit voltage, Voc [60].

**Fig. 4:**
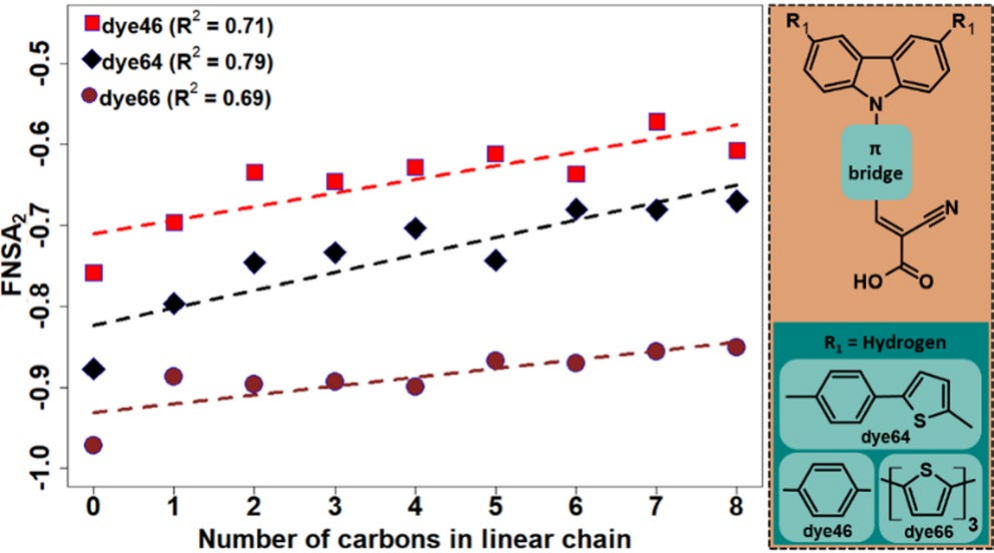
Influence of the length of linear alkyl chains on $\:{\:FNSA}_{2}$

To observe the effect of altering the length of linear alkyl chains on the descriptor $\:{\:\mathrm{F}\mathrm{N}\mathrm{S}\mathrm{A}}_{2}$, we performed symmetrical substitutions on the donor group of three dyes (dye46, dye64, and dye66). In this way, it is possible to observe the relationship between the increase in chain length and the descriptor $\:{\:\mathrm{F}\mathrm{N}\mathrm{S}\mathrm{A}}_{2}\:$(Fig. 4), which supports the adoption of such chains for improving the predicted efficiency of DSSCs.

**Fig. 5:**
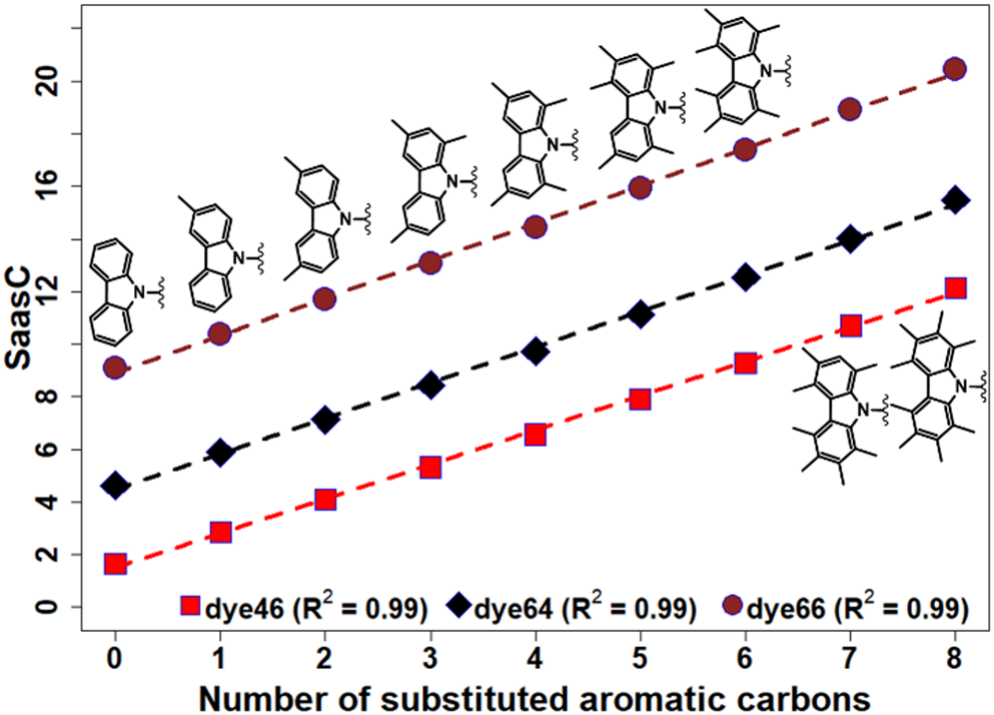
Variation of the $\:SaasC$ descriptor as a function of the number of methyl groups present in the donor group of the dye46, dye64, and dye66 structures

Furthermore, the model also indicates probable positions at which substitutions may negatively impact the predicted PCE. This is quantified by the $\:\mathrm{S}\mathrm{a}\mathrm{a}\mathrm{s}\mathrm{C}$ descriptor, an electrotopological value derived from substituted aromatic carbons. Specifically, $\:\mathrm{S}\mathrm{a}\mathrm{a}\mathrm{s}\mathrm{C}$ is defined as the sum of the E-State values of the substituted aromatic carbons [65, 66]. Since $\:\mathrm{S}\mathrm{a}\mathrm{a}\mathrm{s}\mathrm{C}$ is always a positive value, an increase in the number of substituted aromatic positions leads to a decrease in the predicted PCE. Empirical tests with dyes dye46, dye64, and dye66 revealed a strong linear relationship with increasing methyl substituents on their donor groups (Fig. 5). Although the descriptor thus correlates aromatic substitution with DSSC predicted efficiency, it is important to emphasize its dependence on substituent electronegativity, since the E-state of an atom depends on neighboring groups [68, 69]. That is, the use of substituents with electronegative atoms (e.g., nitrogen and oxygen) will attenuate the effect of aromatic position substitution [68, 69]. In this sense, long-chain alkoxy groups are good substituent options, as they have proven to be efficient for other sensitizer groups [70, 71].

Two additional electrotopological descriptors, $\:\mathrm{m}\mathrm{i}\mathrm{n}\mathrm{H}\mathrm{B}\mathrm{i}\mathrm{n}\mathrm{t}8$ and ndO, are also significant. The $\:\mathrm{m}\mathrm{i}\mathrm{n}\mathrm{H}\mathrm{B}\mathrm{i}\mathrm{n}\mathrm{t}8$ descriptor is defined as the lowest E-state value of atoms involved in potential intramolecular hydrogen bonds at a distance of eight bonds. This descriptor is significance stems from the fact that E-state values tend to decrease when more electronegative species are present [68, 69]. The ndO descriptor, conversely, quantifies the number of oxygen atoms with double bonds within the molecule. A higher ndO value suggests the existence of multiple potential adsorption configurations on the semiconductor surface, which may substantially influence electron injection efficiency into the conduction band. On the other hand, the autocorrelation descriptors ($\:ATS6m$, $\:ATSC6i$, $\:AATSC5s$, $\:MATS4s$, and $\:GATS7c$) describe the distribution of specific properties, such as electronegativity, charge, mass, volume, across a molecule with N atoms [72]. In general, these descriptors indicate that an increase in dye mass or volume often leads to a larger solvent‑accessible surface area, and the presence of atoms with higher ionization energies or greater electronegativity is significantly important for predicting enhanced PCE.

Furthermore, the M-3 model presents four descriptors derived from the eigenvalues of specific matrices: $$\:VE{3}_{Dt},$$$$\:SpMin{1}_{Bhi}$$, $$\:{BCUTc}_{1l}$$, and $$\:VE{2}_{Dzv}$$ [72]. For example, $$\:VE{3}_{Dt}$$ is obtained by summing the coefficients of the last eigenvector of the detour matrix. Thus, $$\:VE{3}_{Dt}$$ has identical values for similar structures, such as dye39 and dye40 (9.34), but shows considerable variations when there is an increase in molecular branching, as seen with dye29 (− 12.54), dye30 (− 6.21), and dye87 (− 6.38) and dye88 (− 2.77). In contrast, the $$\:SpMin{1}_{Bhi}$$ is derived from the absolute value of the smallest eigenvalue of the Burden matrix, weighted by the first ionization potential [72]. Thus, variations in $$\:SpMin{1}_{Bhi}$$ are expected to be correlated with the ionization potential of the sensitizer. Additionally, the descriptor $$\:{BCUTc}_{1l}$$, which corresponds to the smallest eigenvalue of the Burden matrix weighted by partial charges, further aligns it with the concepts presented by the autocorrelation descriptors, especially with $$\:GATS7c$$.  

**Fig. 6:**
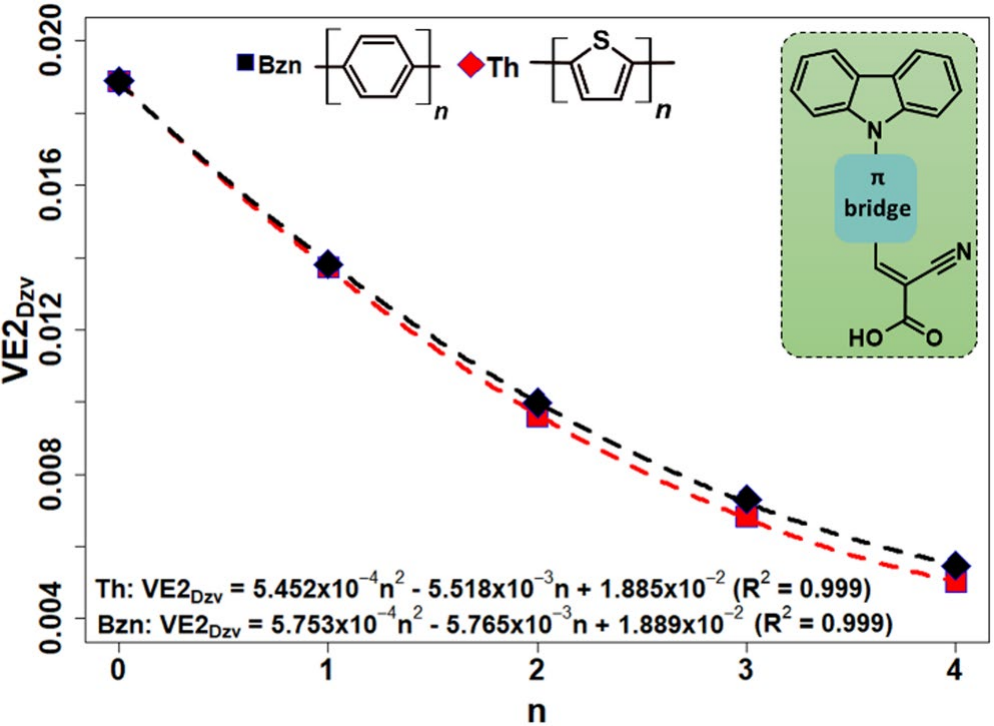
Variation of the $\:VE{2}_{Dzv}$ descriptor as a function of the number of rings present in the π-bridge

Finally, the descriptor $\:VE{2}_{Dzv}$ is calculated as the average of the coefficients of the last eigenvector of the Barysz matrix, weighted by van der Waals (vdW) volumes. Since this descriptor is obtained by weighting with the van der Waals volume, it is expected that modifications in molecular volume will promote a reduction in the $\:VE{2}_{Dzv}$ value. In this sense, to observe this effect, a small experiment was carried out to assess the effect of progressively elongating the π-bridge via thiophene and benzene rings on $\:VE{2}_{Dzv}$ (Fig. 6). Thus, it is notable that changing the π-bridge length can undoubtedly lead to a favorable reduction of this descriptor. That is, species with longer π-bridges tend to exhibit higher predicted PCEs. Therefore, molecular modifications via π-bridge elongation corroborate the need for satisfactory absorption in the visible region and considerable overlap with the solar radiation spectrum, favoring an increase in Jsc.

In general, the M-3 model demonstrates a similar performance to the models proposed in the literature [73]. From the analysis of its descriptors, it is also possible to observe similarities with structural features indicated by the models, such as the presence of alkyl chains to reduce π-π interactions. However, the M-3 model also introduces several new characteristics, such as absorption in the visible region, substitution of aromatic carbons, and the length of the π-bridge. In summary, our best model provides complementary information regarding important structural features for a carbazole-based sensitizer to perform adequately in a DSSC.

### Applying the M-3 model: predictions, molecular modifications and DFT calculations

As previously indicated by the validation metrics applied to the training and test sets, the M-3 model was identified as the best-performing one. Consequently, this model is expected to exhibit satisfactory predictive capability when applied to the assessment of new carbazole-based dyes. To evaluate this critical aspect, the M-3 model was tested on eight novel dye molecules (Table 3) selected from the literature [74–77], where these molecules were selected following the same criteria described in “Dye structures and conversion efficiencies” Section. Thus, after performing the necessary steps to obtain the molecular descriptors (“Structural optimization of dyes and Obtaining molecular descriptors” Sections), the PCE for these eight sensitizers was predicted.

Therefore, it is noteworthy that the predicted PCE values show considerable agreement with their respective experimental values (Table 3) – for these structures, the AD is presented in the Supplementary Material. The only exception is CB-K2K1S, which exhibits an error of approximately 35%. This discrepancy indicates that CB-K2K1S and, likely, its derivatives are not well predicted by the M-3 model. Despite this outlier, the predicted PCEs are strongly correlated with the experimental values—with a Pearson correlation coefficient, r, of approximately 0.75. This result demonstrates the model’s good predictive capability for estimating the PCE of novel carbazole-based sensitizers.

**Table 3:**
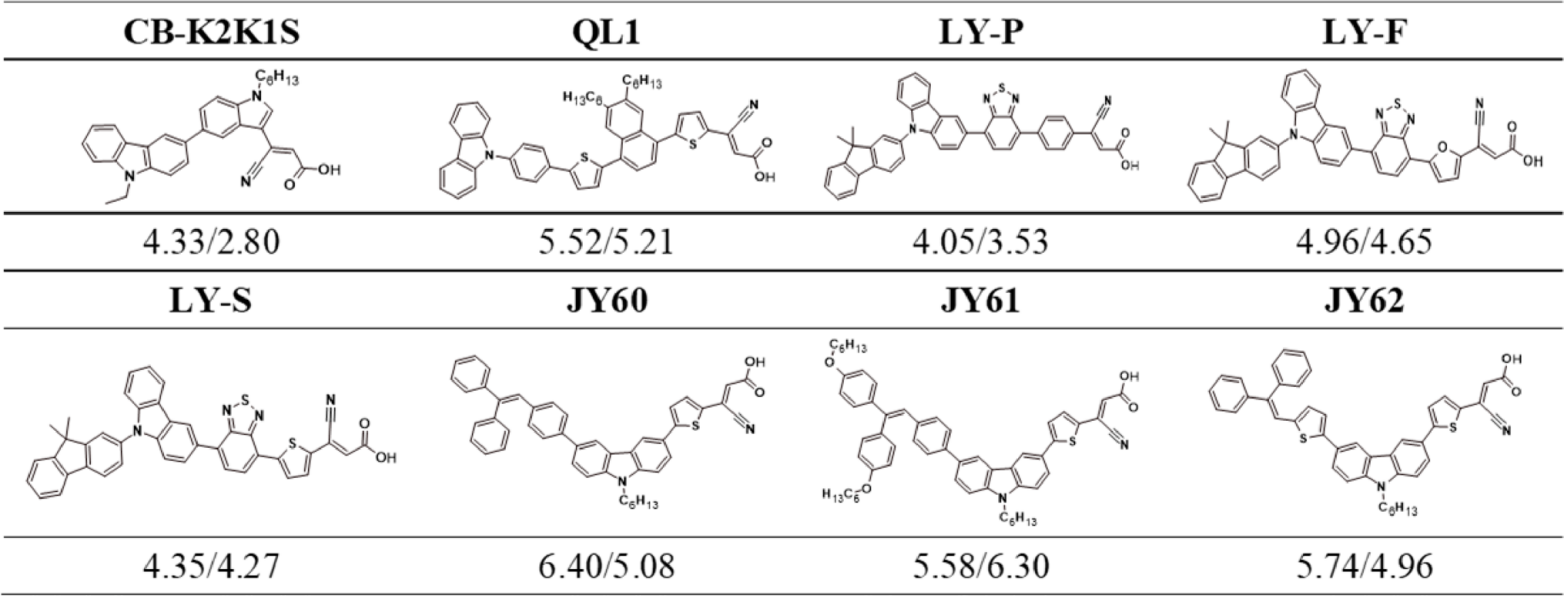
External set of sensitizers employed to evaluate the predictive capability of the models and their experimental/predicted PCE (%)

**Fig. 7:**
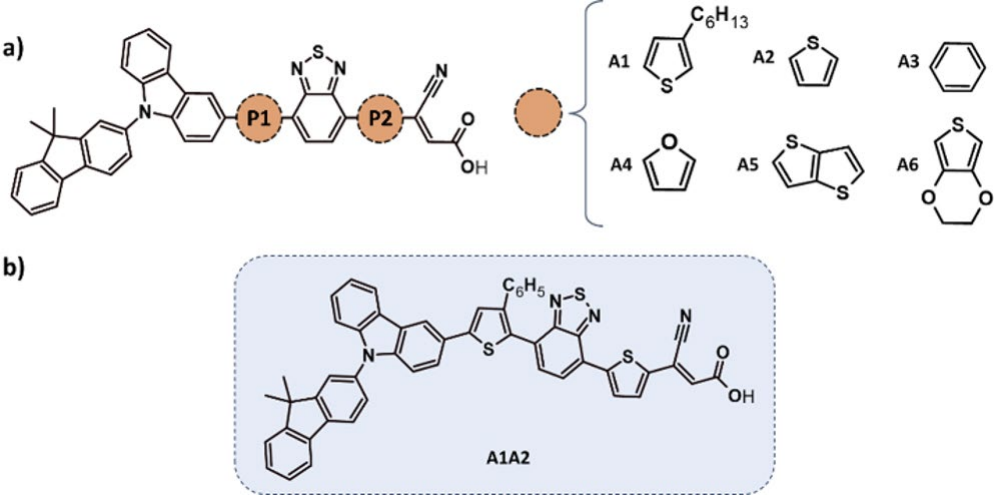
Molecular structure of **a** π-bridge ring systems and their molecular positioning, and **b** an example of a double insertion of rings A1 and A2

Given the strong predictive capability of the M-3 model, we employed it to evaluate new structures obtained through structural modifications, which were informed by insights from the molecular descriptor analysis (“Interpretation of molecular descriptors” Section). Specifically, the modifications were guided by key factors identified by the M-3 model: (i) increased molecular branching; (ii) π-bridge modification; and/or (iii) incorporation of atoms with higher ionization potentials. Therefore, we strategically focused these modifications exclusively on the π-bridge of the LY-P, LY-F, and LY-S dyes, yielding a set of optimized LY sensitizer derivatives (see Fig. 7). This strategy was based on the following: (1) The predicted PCE values for the LY series (Table 3) show good agreement with the experimental values, indicating that predictions for their derivatives are likely to be reliable, (2) LY sensitizers can be synthesized through a few high-yielding steps [75], implying that their derivatives would also be synthetically accessible, and (3) π-bridge modifications have been shown to have greater influence on DSSC efficiency [9, 78–80].

Therefore, we systematically explored these modifications using two molecular positions (P1 and P2) and six established ring systems (A1-A6), all commonly employed in DSSCs. The fused ring in the π-bridge was retained due to its proven efficacy in improving sensitizer performance [19, 81] (Fig. 7). Consequently, this strategy yielded 36 novel potential sensitizers. For each of them, the structure was optimized, and the molecular descriptors required for PCE prediction were calculated.

**Fig. 8:**
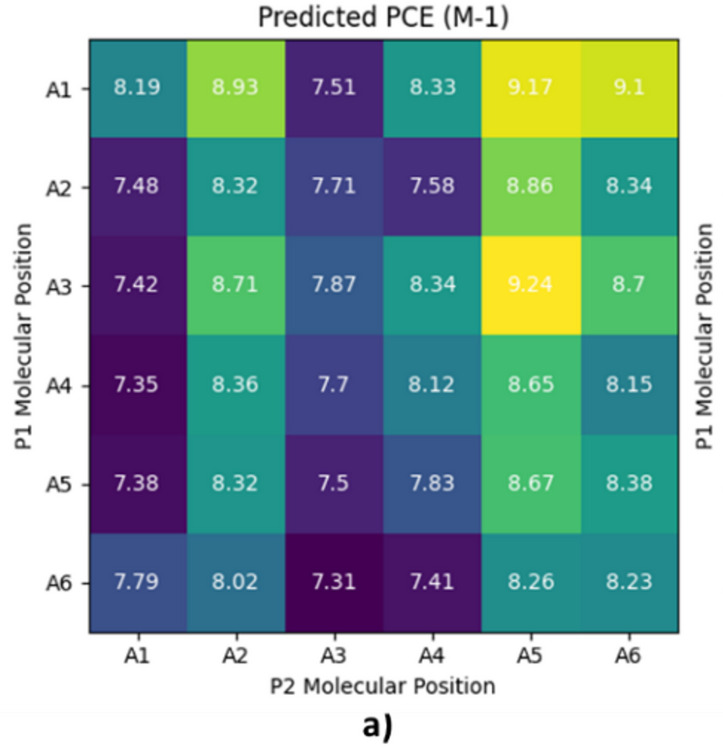
Heatmaps to predicted PCE by the model M-3

The model estimates that all 36 modifications lead to a probable improvement in efficiency (Fig. 8) compared to the initial LY sensitizers (LY-S, LY-P, and LY-F). When examining only the derivatives of LY-S, LY-P, and LY-F, which incorporate the A2, A3, and A4 at the P2 position, the model suggests that modifications with A1 and A2 rings at the P1 position tend to yield higher efficiencies. Furthermore, benzene (A3) appears to be a poor choice for π-bridge, particularly when adopted at P2. Moreover, when the thiophene with a linear alkyl group (A1) occupies the P1 position—a modification that increases molecular branching—the predicted PCE is consistently higher than that of other configurations, particularly in combination with the A2, A5, and A6 rings. These modified structures (A1A2, A1A5 and A1A6) demonstrate nearly twice the predicted efficiency of the unmodified parent molecules (LY-S, LY-P, and LY-F).

In a sense, the very good predicted efficiencies of those molecules agree with several structural features—particularly π-bridge length and enhanced molecular branching—as identified through molecular descriptor analysis and supported by existing experimental studies. However, experimental validation is still necessary to effectively confirm the conversion efficiency for the all 36 proposed molecules, especially for A1A2, A1A5, and A1A6, whose predicted PCEs are around 9%.

However, to corroborate that the proposed modifications can enhance cell efficiency, we conducted DFT and TD‑DFT calculations, which are commonly employed for evaluating a small sample among the newly designed sensitizer structures [82–85]specifically, we compared a set of electronic and spectroscopic properties of the reference sensitizers (LY-S, LY-P, and LY-F) with the three modified structures with the highest predicted PCEs (A1A2, A1A5, and A1A6). Basically, these three structures were selected simply because they exhibit the highest predicted PCEs (~ 9%) by the M-3 model (see Fig. 8). For this comparison, we followed a common computational approach for the theoretical assessment of sensitizers (see Supplementary Material).

Among these properties, the first to be evaluated are the energies and distribution of the frontier molecular orbitals. For proper DSSC function, the HOMO energy is expected to be lower than the redox potential of the $\:{I}_{3}^{-}/{I}^{-}$ electrolyte (− 4.8 eV), while the LUMO energy should be higher than the conduction band of TiO_2_ (− 4.0 eV) [62]. Indeed, both the original and modified structures satisfy these energy level requirements. On the other hand, these six structures exhibit an increase in the HOMO-LUMO gap in the order: A1A2 < A1A5 < LY-S < A1A6 < LY-F < LY-P. Therefore, given that a reduction in the sensitizer’s HOMO-LUMO gap is commonly associated with improved cell performance [86], this provides a further indication that the modified structures could lead to more efficient DSSCs, particularly structures A1A2 (2.18 eV) and A1A5 (2.17 eV).

**Fig. 9:**
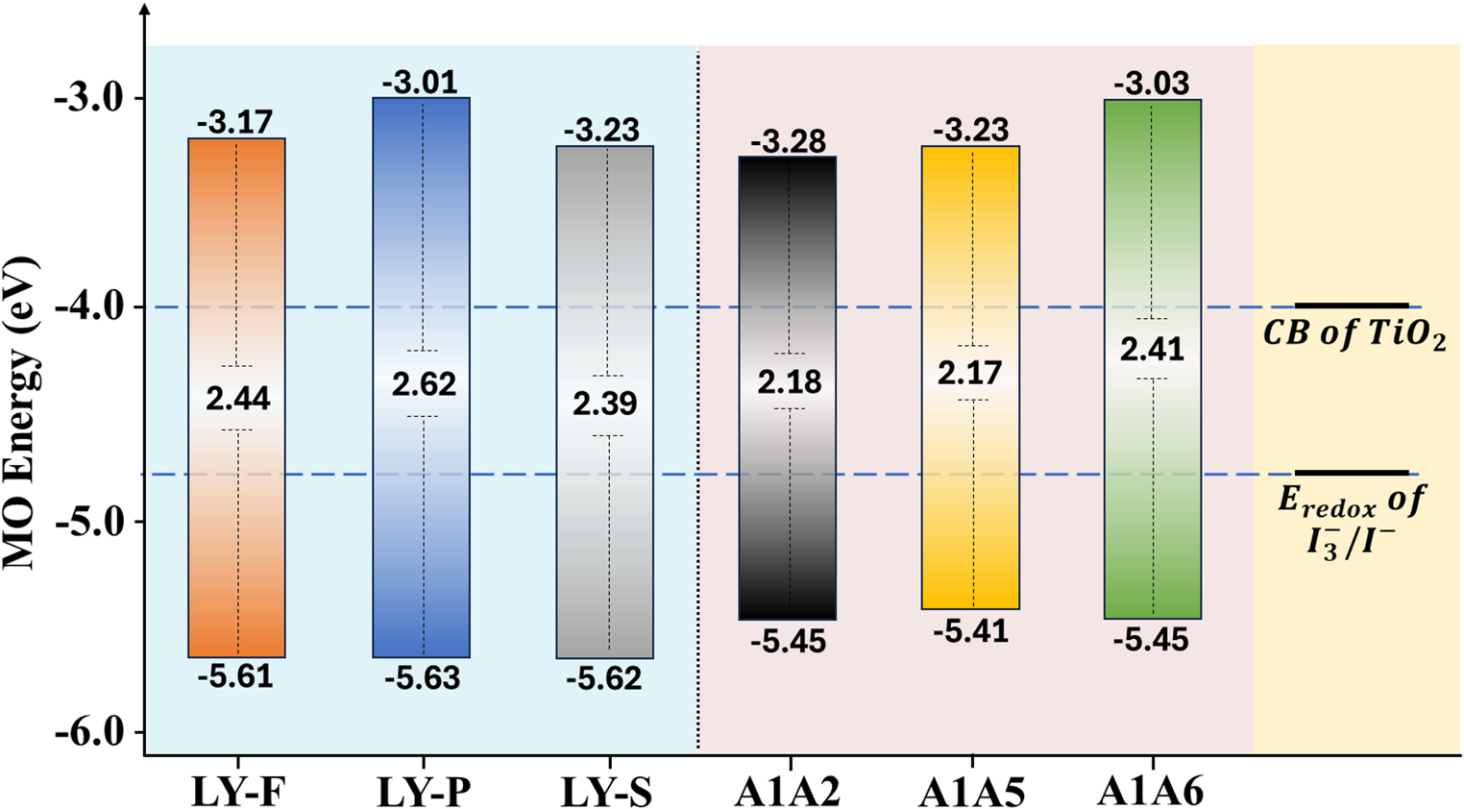
HOMO and LUMO energy levels of the dyes calculated at the B3LYP/6-311G(d, p) level

**Fig. 10:**
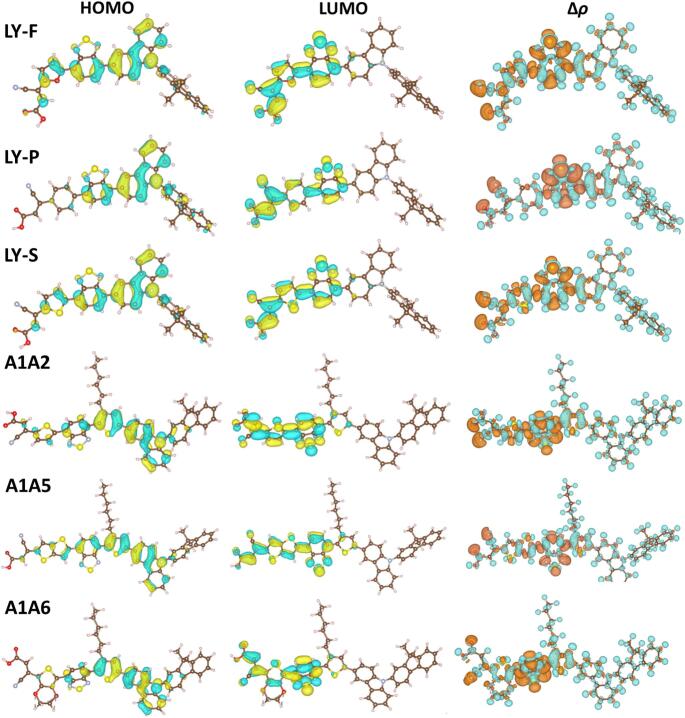
Frontiers molecular orbitals of HOMO and LUMO of the free dyes and the density difference between the ground and excited states (Δρ), where the colors orange and blue represent the electron accumulation and depletion, respectively

In addition to the energy levels of these molecular orbitals, the spatial distribution of electron densities for the free dye also indicates a potential performance enhancement in the modified structures (Fig. 10). Given that the HOMO → LUMO transition is the primary contributor to the maximum absorption at λ_max_ (see Table 4), significant involvement of these orbitals in the intramolecular charge transfer (ICT) process can be anticipated. Consequently, since the rings closest to the acceptor group, and the acceptor group itself, in the modified structures contribute less to the HOMO, an improved charge separation associated with the ICT is expected. This improvement in charge separation is corroborated by the charge density difference maps between the ground and excited states (Fig. 10), Δρ, which show an increase in electron density near the acceptor group and a decrease near the donor group. Therefore, the enhanced ICT would facilitate more efficient charge injection into the semiconductor and a reduction in recombination processes, thereby increasing cell efficiency [82–85].

Indeed, there are clear indications that the modified structures likely exhibit improvements in both electron injection and recombination processes. This is supported by thermodynamic data (Table 4), where the free energy of injection ($\:{\Delta\:}{\mathrm{G}}_{\mathrm{i}\mathrm{n}\mathrm{j}}$) and recombination ($\:{\Delta\:}{\mathrm{G}}_{\mathrm{r}\mathrm{e}\mathrm{c}}$) both show negative values, confirming their thermodynamic favorability. Injection favorability, $\:{\Delta\:}{\mathrm{G}}_{\mathrm{i}\mathrm{n}\mathrm{j}}$, increases according to the order A1A6 < LY-P < A1A2 < A1A5 < LY-F < LY-S. Conversely, recombination favorability, $\:{\Delta\:}{\mathrm{G}}_{\mathrm{r}\mathrm{e}\mathrm{c}}$, follows the order LY-P < LY-S < LY-F < A1A2 ≈ A1A6 < A1A5. Since a more negative free energy value corresponds to a more favorable process, the modified structures tend to both inject electrons slightly more efficiently into the TiO_2_ conduction band and reduce the rate of dye-electron recombination. However, LY-P presents a particular case: despite possessing an injection tendency ($\:{\Delta\:}{\mathrm{G}}_{\mathrm{i}\mathrm{n}\mathrm{j}}$ = − 1.38 eV) comparable to the modified structures, it exhibits one of the highest rates of dye-electron recombination in the set. This vulnerability to significant competition between injection and recombination processes adversely affects the overall cell efficiency.

Conversely, while regeneration remains favorable ($\:{\Delta\:}{\mathrm{G}}_{\mathrm{r}\mathrm{e}\mathrm{g}}$ < 0), the structural modification is accompanied by small increase in the $\:{\Delta\:}{\mathrm{G}}_{\mathrm{r}\mathrm{e}\mathrm{g}}$ values (Table 4), indicating marginally less efficient regeneration of their oxidized state. Nevertheless, since regeneration processes occur on the nanosecond timescale [87], the A1A2, A1A6, and A1A5 species are still expected to undergo sufficiently rapid regeneration to ensure satisfactory energy conversion. Additionally, the proposed structures exhibit favorable changes in the absorption wavelength, λ_max_, and their corresponding oscillator strength, *f*, which increase in the order LY-P < A1A6 < LY-F < LY-S < A1A2 < A1A5. This increase suggests that the modified structures generally display more intense, red-shifted absorptions, leading to a greater overlap with the solar spectrum and more intense absorption of incident radiation.

**Table 4:** Parameters from charge transfer, free energies, $\:{\Delta\:}{\mathrm{G}}_{\mathrm{i}\mathrm{n}\mathrm{j}/\mathrm{r}\mathrm{e}\mathrm{g}/\mathrm{r}\mathrm{e}\mathrm{c}}$ (eV) and wavelength maximum absorption, λ_max_, and its oscillator strength and main composition

Molecule	$\Delta {\mathrm{G}}_{{{\mathrm{inj}}}}$	$\Delta {\mathrm{G}}_{{{\mathrm{rec}}}}$	$\Delta$ G_reg_	λ_max_	f	Main composition
LY-F	− 1.02	− 1.61	− 0.81	471.45	1.15	H-1 → L (14.36) H → L (72.72)
LY-P	− 1.38	− 1.63	− 0.83	412.86	1.08	H → L (52.22) H → L + 1 (21.00)
LY-S	− 0.98	− 1.62	− 0.82	475.10	1.32	H-1 → L (15.24) H → L (69.15)
A1A2	− 1.08	− 1.45	− 0.65	490.69	1.33	H-1 → L (23.80) H → L (58.97)
A1A5	− 1.05	− 1.41	− 0.61	505.34	1.75	H-1 → L (19.59) H → L (63.39)
A1A6	− 1.36	− 1.45	− 0.65	442.10	1.14	H-1 → L (25.78) H → L (47.82)

Beyond the isolated dyes, we also calculated some properties related to the interaction between the dyes and the semiconductor. It is noteworthy that all dyes exhibit a favorable binding energy ($\:{\mathrm{E}}_{\mathrm{b}\mathrm{i}\mathrm{n}\mathrm{d}}<\:0$) for the formation of the Dye@(TiO_2_)_9_ cluster (Table S3). Furthermore, the Ti-O bond lengths and $\:{\mathrm{E}}_{\mathrm{b}\mathrm{i}\mathrm{n}\mathrm{d}}$ values show considerable similarity across all systems, demonstrating the minimal influence of the structural modifications on these particular properties. Regarding the frontier molecular orbitals of these complexes (Figure S1), the HOMO is distributed over the donor moiety and along the π-bridge, while the LUMO is localized on the (TiO_2_)_9_ cluster. This distribution reinforces the occurrence of efficient charge injection into the TiO_2_ following dye excitation. In addition, the formation of the cluster is accompanied by: (i) a stabilization of the LUMO and a consequent narrowing of the HOMO-LUMO gap (Table S2), with the smallest gaps presented by the systems formed with A1A2, A1A5, and A1A6; and (ii) a subtle red-shift in λ_max_, along with a considerable increase in absorption intensity (Table S3).

Thus, through these DFT calculations, which are common in the theoretical evaluation of new sensitizers, a favorable tuning of the electronic and spectroscopic properties associated with high-performance DSSC sensitizers is evident. In other words, the DFT approach corroborates the fact that the proposed modifications can enhance cell efficiency, as initially indicated by the model. Therefore, the M-3 model can be reliably employed for the rapid screening of carbazole-based sensitizers.

## Conclusions

QSPR modeling was employed with a set of carbazole-based dyes to predict the power conversion efficiencies (PCEs) of dye-sensitized solar cells (DSSCs). The methodology used for model development resulted in three models (M-1, M-2, and M-3) that met established internal and external validation criteria for QSPR models, providing good fits, satisfactory prediction ability, and robustness. The most predictive model (M-3) identified key structural characteristics associated with higher PCE, including well-established parameters such as visible light absorption, as well as other subtler aspects, like insertion of alkyl chains in positions that could minimize the π-π interactions. Furthermore, from 36 molecular modifications to the π-bridge, the model identified three structures with predicted PCE approaching 9%, representing a potential improvement in power conversion efficiency when compared to the reference sensitizers. However, experimental validation to confirm the efficiency of structures A1A2, A1A5, and A1A6 is still necessary. Moreover, DFT and TD-DFT calculations on a select sample of the modified structures reveal a favorable tuning of the electronic and spectroscopic properties that are critical for high DSSC performance. Thus, these linear models enable rapid and cost-efficient prediction of PCE for promising new carbazole‑based dye candidates only, since only carbazole‑based dyes were used to build the models.

## Supplementary Information

Below is the link to the electronic supplementary material.

Supplementary Material 1

Supplementary Material 2

Supplementary Material 3

## Data Availability

Data available in: https://github.com/e-mattos/New_Carbazole_lin_models.
