# Supplementary material for: A linear models approach to optimize carbazole-based dyes for solar cell applications
Source: J Comput Aided Mol Des. 2026 Jul 6;40(1):174. doi: 10.1007/s10822-026-00874-7 (PMC13337791; doi:10.1007/s10822-026-00874-7)
Supplement: Supplementary file 3 — Supplementary Material 3 [file 10822_2026_874_MOESM3_ESM.pdf]

A LINEAR MODELS APPROACH TO OPTIMIZE CARBAZOLE-BASED DYES FOR SOLAR CELL APPLICATIONS

Emanuel F. dos S. Mattos[a], *Carlos R. A. Daniel*[b], *Nivan B. da Costa Júnior*[a]*

[a]Department of Chemistry, Federal University of Sergipe Foundation, Sergipe, 49100-000, Brazil

[b]Department of Statistics, Federal University of Sergipe Foundation, Sergipe, 49100-000, Brazil

*Corresponding Author: **nivan@academico.ufs.br**

Applicability Domain for the New Species

For a new species i, we consider that it belongs to the applicability domain (AD) if its leverage h_i is lower than the leverage limit h*. More precisely, we calculate the leverage of a given species as $h_i = x_i(X^T X)^{-1} x_i^T$ Thus, for each model, the species of the external set (Table 3) fall within the AD of each model, yielding reasonably reliable predictions (Figure S1 a–c). On the other hand, the set of structural modifications (Figure 7) falls outside the applicability domain only for Model M-3 (Figure S1 d–f).

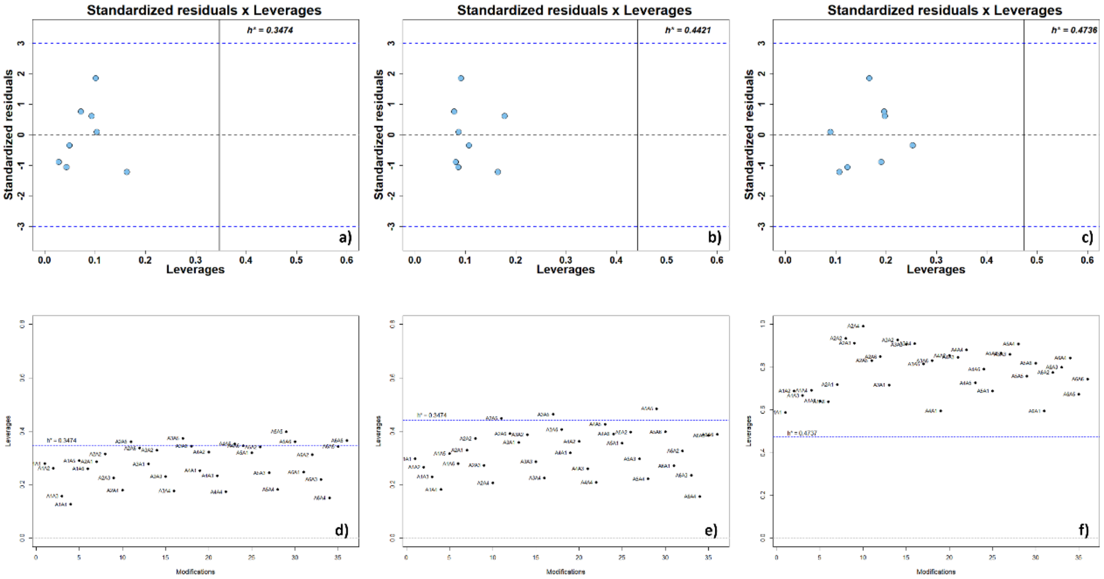

Figure S1. AD for the external sets (a–c) and molecular modifications (d–f)

However, upon observing the predictions associated with the three structures selected in the main text (A1A2, A1A5, and A1A6) for the other two models (Figure S2), it is notable that the predicted PCEs are indeed ~9%. This does not invalidate the conclusion of a probable improvement in PCE associated with these modifications.

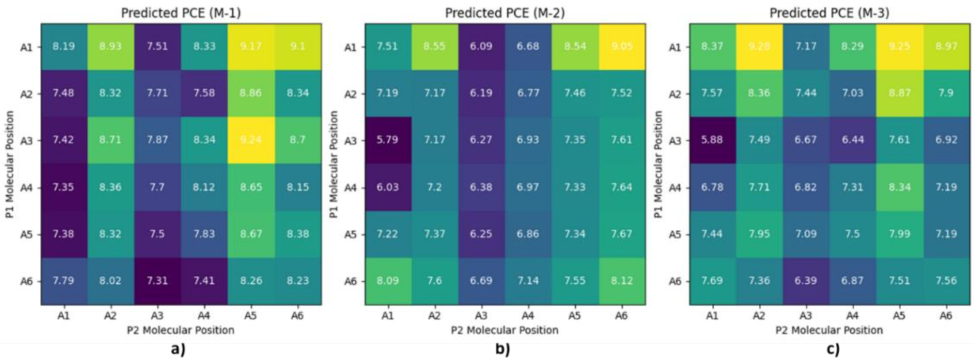

Figure S2. Heatmaps to predicted PCE by the three models.

DFT approach to selected modified dyes

The molecular structures of all molecules were optimized using Density Functional Theory (DFT) with the B3LYP functional and the 6-311G(d, p) basis set. Based on these optimized structures, the first 15 excited states were calculated using the CAM-B3LYP functional and the same basis set employed for the optimization. The ground and excited states for all molecules were obtained considering the solvent effect (chloroform) using the Conductor-like Polarizable Continuum Model (CPCM).

To simulate the interaction between the dyes and the semiconductor, we used the $(TiO_2)_9$ cluster due to its proven utility in other computational studies [1–5]. We considered the same bidentate binding mode because of its frequently reported stability [2–4]. Subsequently, the Dyes@$(TiO_2)_9$ systems were optimized using the B3LYP functional with the 6-311G(d, p) and LANL2DZ basis sets, applying the latter exclusively to Ti atoms. The first 15 excited states were then calculated using the CAM-B3LYP functional and the two aforementioned basis sets. In addition, the solvent effect (chloroform) was considered in both calculations via the CPCM model.

Parameters related to photovoltaic properties

In DSSC research, it is notable that a significant amount of work has employed computational methods to evaluate potential new sensitizers, with Density Functional Theory (DFT) being the most frequent approach. This method is commonly used to explore parameters related to DSSC performance, such as J_{SC} and V_{OC} [6–8]. For instance, J_{SC} (Eq. S1) is related to the elementary charge, q, charge collection efficiency, η_{coll}, electron injection efficiency, η_{inj}, light-harvesting efficiency, $LHE(\lambda)$, and the photon flux from incident radiation, $I_S(\lambda)$.

$$J_{SC} = q\int LHE(\lambda) \times \eta_{inj} \times \eta_{coll} \times I_S(\lambda) d\lambda \qquad \text{Eq. S1}$$

In this equation, the $LHE(\lambda)$ and η_{inj} can be evaluated by DFT methods. For example, a commonly employed approximation for $LHE(\lambda)$ involves only the oscillator strength, f, associated with the maximum absorption of the dye (Eq. S2).

$$LHE = 1 - 10^{-f} \qquad \text{Eq. S2}$$

On the other hand, because directly estimating η_{inj} requires obtaining kinetic parameters related to charge injection and intramolecular decay [9,10], this term is commonly

assessed indirectly via the injection free energy, ΔG_{inj}, given their well-established satisfactory relationship $\left(\eta_{inj} \propto \Delta G_{inj}\right)$ [9,10]. In this regard, ΔG_{inj} is readily obtained since it depends exclusively on the conduction band energy of the semiconductor, E_{CB} (-4.0 eV for TiO_2), and the oxidation potential of the dye in the excited state, E^*_{Dye} (Eq. S3). Where E^*_{Dye} depends on the vertical excitation energy for the maximum absorption, $E_{\lambda_{max}}$, and the oxidation potential of the dye in the ground state, E_{Dye}, which is essentially the negative of the HOMO energy (Eq. S4).

$$\Delta G_{inj} = E^*_{Dye} - E_{CB} \qquad \text{Eq. S3}$$

$$E^*_{dye} = E_{Dye} - E_{\lambda_{max}} \qquad \text{Eq. S4}$$

A parameter related to the regeneration of the oxidized dye can also be estimated by obtaining the change in the regeneration free energy, ΔG_{reg}, a commonly used approach expressed by Equation S5. Here, $E_{I_3^-/I^-}$ corresponds to the redox potential of the I_3^-/I^- couple (-4.8 eV).

$$\Delta G_{reg} = E_{I_3^-/I^-} - E_{Dye} \qquad \text{Eq. S5}$$

References

[1] D. Fadili, Z.M.E. Fahim, S.M. Bouzzine, M. Hamidi, Improved photovoltaic performance of phosphonic acid-based sensitized solar cells via an electron-withdrawing moiety: A density of functional theory study, Int J Quantum Chem 121 (2021). https://doi.org/10.1002/qua.26431.

[2] Y. Li, B. Xu, P. Song, F. Ma, M. Sun, D-A-π-A System: Light Harvesting, Charge Transfer, and Molecular Designing, Journal of Physical Chemistry C 121 (2017) 12546–12561. https://doi.org/10.1021/acs.jpcc.7b02328.

[3] M. Gong, L. Zeng, W. Wang, X. Dong, Z. Yu, S. Wang, Y. Yang, Effects of Several Auxiliary Acceptors and Anchoring Groups on Charge Transfer and Photophysical Properties of D-A-π-A Type DSSCs: A DFT Study, J Fluoresc 35 (2025) 2285–2297. https://doi.org/10.1007/s10895-024-03685-x.

[4] J. Zhang, Y.H. Kan, H. Bin Li, Y. Geng, Y. Wu, Z.M. Su, How to design proper π-spacer order of the D-π-A dyes for DSSCs? A density functional response, Dyes and Pigments 95 (2012) 313–321. https://doi.org/10.1016/j.dyepig.2012.05.020.

[5] R. Sánchez-De-Armas, M.Á. San Miguel, J. Oviedo, J.F. Sanz, Coumarin derivatives for dye sensitized solar cells: A TD-DFT study, Physical Chemistry Chemical Physics 14 (2012) 225–233. https://doi.org/10.1039/c1cp22058f.

[6] W. Zhang, L. Wang, L. Mao, J. Jiang, H. Ren, P. Heng, H. Ågren, J. Zhang, Computational Protocol for Precise Prediction of Dye-Sensitized Solar Cell Performance, Journal of Physical Chemistry C 124 (2020) 3980–3987. https://doi.org/10.1021/acs.jpcc.9b10869.

[7] G. Consiglio, A. Gorcynski, S. Petralia, G. Forte, Computational study of linear carbon chain based organic dyes for dye sensitized solar cells, RSC Adv 13 (2023) 1019–1030. https://doi.org/10.1039/d2ra06767f.

[8] D.D. Deng, J.K. Shi, J.X. Tong, F. Wang, X.L. Shi, J.Q. Li, W. Wei, Theoretical insights into single- and co-sensitization of indolocarbazole-based dyes on anatase (101) surface for efficient dye-sensitized solar cells, Comput Theor Chem 1247 (2025). https://doi.org/10.1016/j.comptc.2025.115143.

[9] R. Katoh, A. Furube, Electron injection efficiency in dye-sensitized solar cells, Journal of Photochemistry and Photobiology C: Photochemistry Reviews 20 (2014) 1–16. https://doi.org/10.1016/j.jphotochemrev.2014.02.001.

[10] R. Katoh, A. Furube, T. Yoshihara, K. Hara, G. Fujihashi, S. Takano, S. Murata, H. Arakawa, M. Tachiya, Efficiencies of Electron Injection from Excited N3 Dye into Nanocrystalline Semiconductor (ZrO2, TiO2, ZnO, Nb2O 5, SnO2, In2O3) Films, Journal of Physical Chemistry B 108 (2004) 4818–4822. https://doi.org/10.1021/jp031260g.

[11] M. Hiiti Tsere, R. Costa, G. Deogratias, T. Pogrebnaya, A. Pogrebnoi, R. Machunda, O.S. Al-Qurashi, N. Wazzan, N. Surendra Babu, Effect of Electron Donor Groups on Optoelectronic Properties of Betalain Dyes: A DFT Study, ChemistryOpen 14 (2025). https://doi.org/10.1002/open.202400525.

[12] N. Kungwan, P. Khongpracha, S. Namuangruk, J. Meeprasert, C. Chitpakdee, S. Jungsuttiwong, V. Promarak, Theoretical study of linker-type effect in carbazole–carbazole-based dyes on performances of dye-sensitized solar cells, Theor Chem Acc 133 (2014) 1–14. https://doi.org/10.1007/s00214-014-1523-4.

Others result of free Dyes and Dye@(TiO2)9 systems

Structurally, all optimized complexes exhibit Ti–O bond lengths consistent with those obtained in other reported studies [11,12], falling within the range of 2.0 to 2.2 Å (see Table S1). Furthermore, given the negative energy for the interaction between the sensitizer and the $(TiO2)_9$, E_{bind} (Eq. S6), it follows that the formation of the Dye@$(TiO2)_9$ complex is favorable.

$$E_{bind} = E_{Dye@(TiO2)9} - (E_{Dye} + E_{(TiO2)9}) \qquad \text{Eq. S6}$$

Table S1 – Bond lengths and interaction energy for the formation of the Dye@$(TiO_2)_9$ clusters.

Molecule	Ti-O1	Ti-O2	Energy bind(eV)
**LY-F**	2.192	2.055	-1.384
**LY-P**	2.059	2.245	-1.268
**LY-S**	2.180	2.073	-1.351
**A1A2**	2.071	2.194	-1.261
**A1A5**	2.148	2.096	-1.386
**A1A6**	2.163	2.069	-1.457

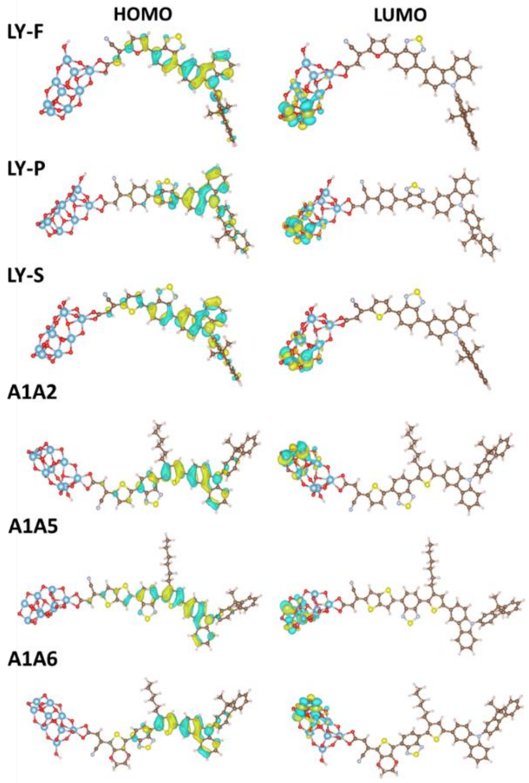

Figure S3. Frontiers molecular orbitals of HOMO and LUMO of the complexes Dye@$(TiO2)_9$.

Table S2 – Energy (eV) of the HOMO. LUMO. and Gap for the Dye@$(TiO_2)_9$ clusters.

Molecule	HOMO	LUMO	*GAP*
**LY-F**	-5.612	-3.455	2.157
**LY-P**	-5.606	-3.515	2.091
**LY-S**	-5.632	-3.464	2.167
**A1A2**	-5.437	-3.514	1.923
**A1A5**	-5.403	-3.478	1.924
**A1A6**	-5.444	-3.445	1.999

Table S3 – Absorption λ_{max}, oscillator strength and main compositions to Dye@$(TiO_2)_9$.

Molecule	λ (nm)	f	Main Compositions*
**LY-F**	482.59	1.405	H-1 → L (15.52%)
			H → L (48.41%)
			H → L+1 (11.71%)
**LY-P**	411.67	1.235	H → L+2 (13.31%)
			H → L+3 (15.02%)
			H → L+8 (14.47%)
			H → L+9 (16.13%)
**LY-S**	464.02	1.530	H-1 → L (12.70%)
			H → L (38.90%)
			H → L+1 (13.83%)
**A1A2**	499.49	1.596	H-1 → L (18.97%)
			H → L (53.43%)
**A1A5**	502.62	2.033	H-1 → L (15.24%)
			H → L (42.14%)
**A1A6**	449.07	1.497	H-1 → L+1 (13.73%)
			H → L+1 (18.00%)
			H → L+2 (10.40%)

* H: HOMO, L: LUMO

Table S5. Dye Structures and Photovoltaic Performances

Dye	%PCE	Sol.	doi	Dye	%PCE	Sol.	doi
**1.**	5.64	Et	10.1016/j.tet.2013.02.058	**33.**	4.11	THF	10.1039/C3TA11748K
**2.**	4.22	Di	10.1021/am404948w	**34.**	6.40	THF	10.1039/C3TA11748K
**3.**	4.95	Di	10.1021/am404948w	**35.**	5.43	THF	10.1039/C3TA01657A
**4.**	6.04	Di	10.1021/am404948w	**36.**	6.50	THF	10.1039/C3TA01657A
**5.**	5.48	Di	10.1021/am404948w	**37.**	2.96	THF	10.1039/C3TA01657A
**6.**	1.21	Ac	10.1108/PRT-09-2014-0077	**38.**	4.61	THF	10.1039/C3TA01657A
**7.**	2.82	Ac	10.1108/PRT-09-2014-0077	**39.**	6.01	Di	10.1021/am508400a
**8.**	3.69	Ac	10.1108/PRT-09-2014-0077	**40.**	6.93	Di	10.1021/am508400a
**9.**	5.92	Ac	10.1016/j.jpowsour.2020.227776	**41.**	7.54	Di	10.1021/am508400a
**10.**	2.39	Tr	doi.org/10.1021/jp1055842	**42.**	3.64	Di	10.1002/ejoc.201300373
**11.**	2.48	Tr	doi.org/10.1021/jp1055842	**43.**	4.80	Di	10.1002/ejoc.201300373
**12.**	7.44	Tr	10.1016/j.electacta.2018.08.068	**44.**	5.69	Di	10.1002/ejoc.201300373
**13.**	3.50	Tr	10.1016/j.dyepig.2016.08.013	**45.**	4.62	Di	10.1002/ejoc.201300373
**14.**	2.68	Ac	10.1246/cl.2010.864	**46.**	1.77	Di	10.1016/j.dye-pig.2012.03.028
**15.**	1.87	Di	10.1002/ejoc.201600353	**47.**	5.13	Di	10.1021/acsami.5b08888
**16.**	4.54	Di	10.1002/ejoc.201600353	**48.**	7.69	Di	10.1021/acsami.5b08888
**17.**	2.52	Di	10.1002/ejoc.201600353	**49.**	3.52	Di	10.1021/jp304489t
**18.**	4.57	Di	10.1002/ejoc.201600353	**50.**	4.10	Di	10.1021/jp304489t
**19.**	2.49	Tr	10.1016/j.sol-mat.2009.11.014	**51.**	5.12	Di	10.1021/jp304489t
**20.**	3.18	Tr	10.1016/j.sol-mat.2009.11.014	**52.**	3.34	Di	10.1016/j.sole-ner.2018.09.073
**21.**	6.60	Tr	10.1016/j.tet.2014.01.001	**53.**	5.98	THF	10.1021/am5067145
**22.**	6.73	Tr	10.1016/j.tet.2014.01.001	**54.**	6.48	Di	10.1016/j.jpowsour.2015.01.148
**23.**	2.17	Tr	10.1039/C6RA01185C	**55.**	7.03	Di	10.1016/j.jpowsour.2015.01.148
**24.**	0.98	Tr	10.1039/C6RA01185C	**56.**	9.20	Di	10.1039/C3TA12368E
**25.**	2.69	Tr	10.1039/C6RA01185C	**57.**	7.15	Di	10.1039/C7NJ04629D
**26.**	0.98	Tr	10.1039/C6RA01185C	**58.**	7.26	Di	10.1039/C7NJ04629D
**27.**	1.11	Tr	10.1039/C6RA01185C	**59.**	0.57	Di	10.1007/s10854-018-9750-4
**28.**	5.78	Tr	10.1016/j.dye-pig.2019.01.033	**60.**	0.92	Di	10.1007/s10854-018-9750-4
**29.**	5.23	Tr	10.1016/j.dye-pig.2019.01.033	**61.**	6.44	Di	10.1016/j.dye-pig.2015.07.034
**30.**	5.97	Tr	10.1016/j.dye-pig.2019.01.033	**62.**	4.77	Di	10.1016/j.dye-pig.2015.07.034
**31.**	6.09	THF	10.1039/C3TA11748K	**63.**	4.38	Di	10.1016/j.dyepig.2015.09.004
**32.**	5.55	THF	10.1039/C3TA11748K	**64.**	2.74	Di	10.1016/j.dye-pig.2013.09.025

**65.**	2.94	Di	10.1016/j.dyepig.2013.09.025	**96.**	5.23	THF	10.1016/j.dyepig.2018.06.010
**66.**	4.30	Di	10.1016/j.dyepig.2013.09.025	**97.**	5.30	THF	10.1016/j.dyepig.2018.10.004
**67.**	4.86	Di	10.1016/j.dyepig.2013.09.025	**98.**	5.65	THF	10.1039/C3RA43057J
**68.**	6.25	Di	10.1016/j.jpowsour.2016.04.043	**99.**	6.23	THF	10.1039/C3RA43057J
**69.**	8.09	Di	10.1016/j.jpowsour.2016.04.043	**100.**	7.15	THF	10.1039/C3RA43057J
**70.**	6.98	Di	10.1016/j.jpowsour.2016.04.043	**101.**	5.20	THF	10.1039/C3RA43057J
**71.**	7.58	Di	10.1016/j.jpowsour.2016.04.043	**102.**	5.82	THF	10.1039/C3RA43057J
**72.**	7.50	Di	10.1039/C3RA22249G	**103.**	6.10	THF	10.1021/ol402931u
**73.**	7.01	Di	10.1039/C9TC01520E	**104.**	5.50	THF	10.1021/ol402931u
**74.**	8.01	Di	10.1039/C9TC01520E	**105.**	5.11	THF	10.1021/ol402931u
**75.**	5.06	Di	10.1039/C9TC01520E	**106.**	4.87	Di	10.1002/gch2.201900034
**76.**	4.23	DMF	10.1039/C7PP00350A	**107.**	4.49	THF	10.1039/C3TA12901B
**77.**	5.97	DMF	10.1039/C7PP00350A	**108.**	4.60	THF	10.1039/C3TA12901B
**78.**	5.34	DMF	10.1039/C7PP00350A	**109.**	3.03	THF	10.1002/asia.201402654
**79.**	5.02	Et	10.1016/j.tet.2006.12.082	**110.**	5.9	Tr	10.1039/C4QO00285G
**80.**	5.15	Et	10.1016/j.tet.2006.12.082	**111.**	6.5	Tr	10.1039/C4QO00285G
**81.**	3.87	Et	10.1016/j.tet.2006.12.082	**112.**	7.0	Tr	10.1039/C4QO00285G
**82.**	3.76	Et	10.1016/j.tet.2006.12.082	**113.**	4.31	Et	10.1021/jp906334w
**83.**	7.1	Et	10.1039/C5TA06548H	**114.**	5.96	Tr	10.1016/j.tet.2015.04.018
**84.**	8.48	Met	10.1016/j.dyepig.2018.03.072	**115.**	5.2	Di	10.1016/j.dyepig.2015.02.020
**85.**	4.69	Met	10.1016/j.dyepig.2018.03.072	**116.**	6.5	Di	10.1016/j.dyepig.2015.02.020
**86.**	4.65	THF	10.1016/j.dyepig.2012.10.002	**117.**	6.5	Di	10.1016/j.dyepig.2015.02.020
**87.**	3.96	THF	10.1039/C5RA02720A	**118.**	6.95	Di	10.1039/C4TA05162A
**88.**	2.85	THF	10.1039/C5RA02720A	**119.**	6.67	Di	10.1039/C4TA05162A
**89.**	7.52	THF	10.1039/C6TA02275H	**120.**	2.30	Di	10.1021/jo200501b
**90.**	8.51	THF	10.1039/C6TA02275H	**121.**	3.19	Di	10.1016/j.tetlet.2014.04.037
**91.**	7.58	THF	10.1016/j.dyepig.2016.12.013	**122.**	5.10	Di	10.1021/am500947k
**92.**	6.48	THF	10.1016/j.dyepig.2018.06.010	**123.**	4.90	Di	10.1002/cssc.201200975
**93.**	6.33	THF	10.1016/j.dyepig.2018.06.010	**124.**	5.80	Di	10.1002/cssc.201200975
**94.**	7.77	THF	10.1016/j.dyepig.2018.06.010	**125.**	5.80	Di	10.1002/cssc.201200975
**95.**	5.23	THF	10.1016/j.dyepig.2018.06.010	**126.**	5.60	Di	10.1002/cssc.201200975

Ac – Acetonitrile; **Et –** Ethanol; **Di –** Dichloromethane; **DMF -** Dimethylformamide**; Met** – Methanol**; Tr** – Trichloromethane; **THF** – Tetrahydrofuran;

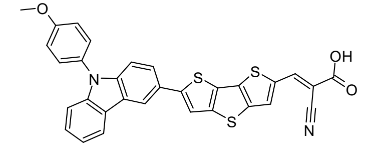 dye1	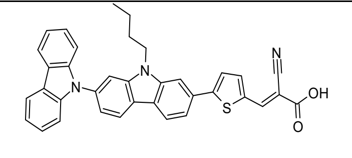 dye2	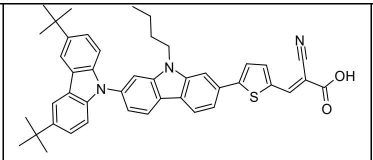 dye3	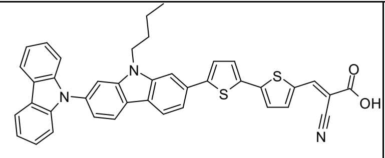 dye4	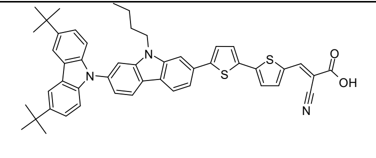 dye5
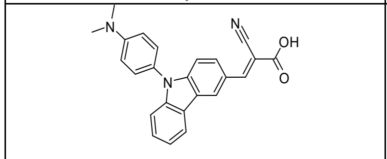 dye6	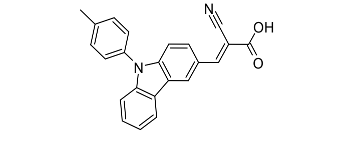 dye7	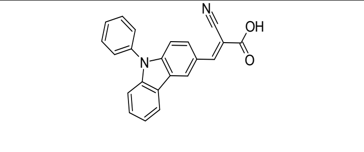 dye8	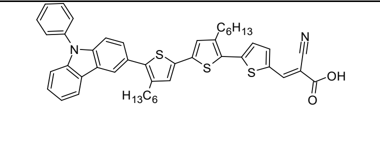 dye9	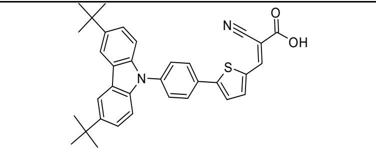 dye10
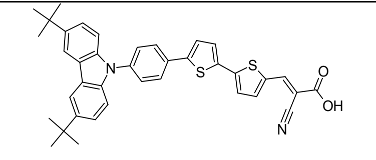 dye11	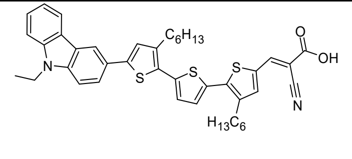 dye12	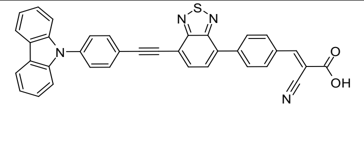 dye13	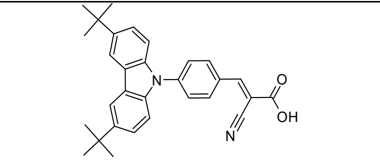 dye14	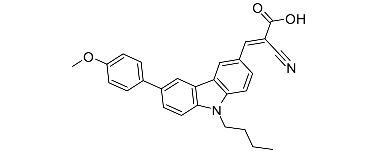 dye15
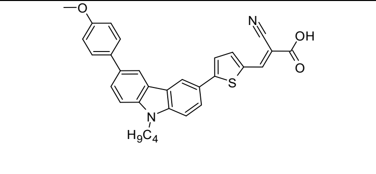 dye16	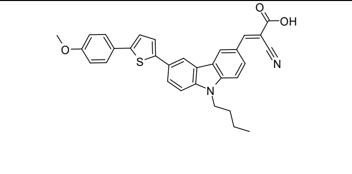 dye17	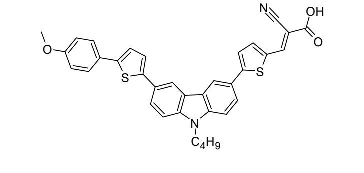 dye18	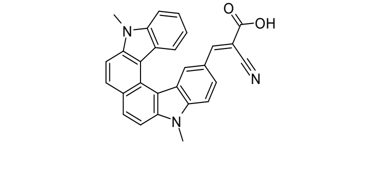 dye19	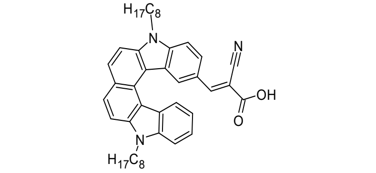 dye20
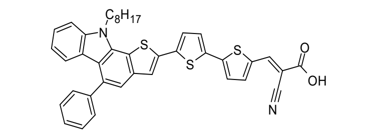 dye21	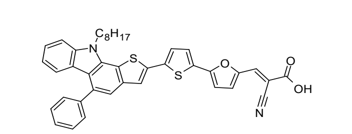 dye22	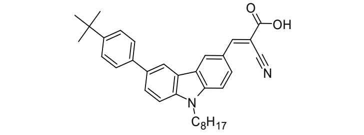 dye23	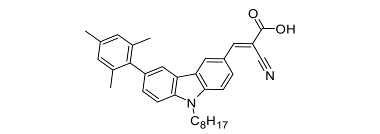 dye24	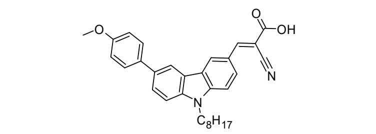 dye25
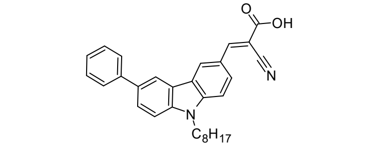 dye26	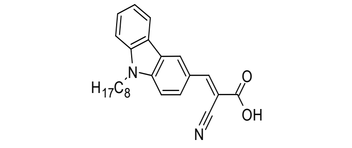 dye27	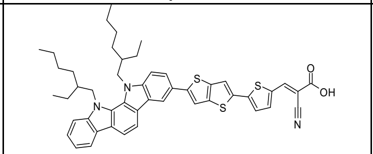 dye28	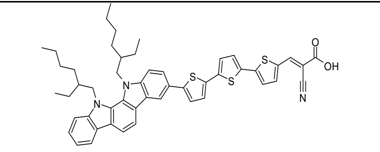 dye29	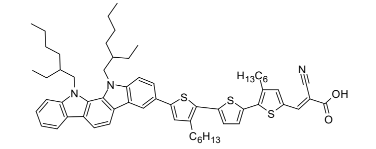 dye30

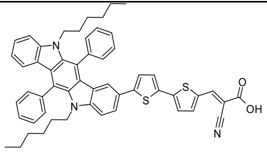 dye31	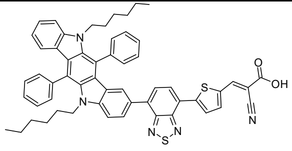 dye32	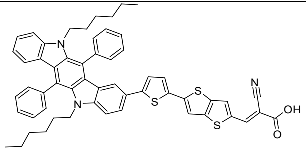 dye33	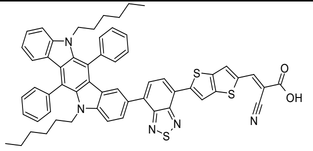 dye34	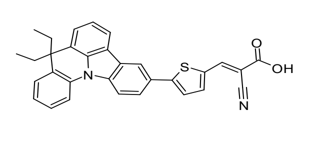 dye35
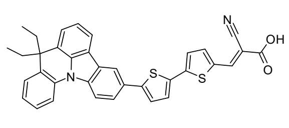 dye36	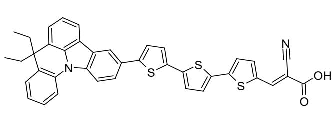 dye37	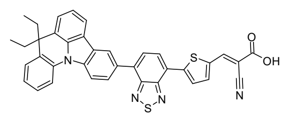 dye38	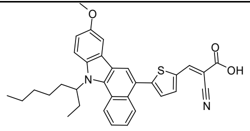 dye39	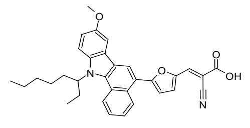 dye40
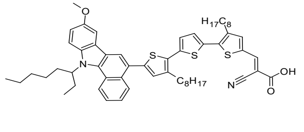 dye41	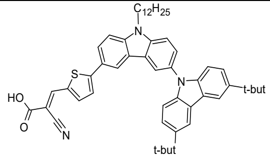 dye42	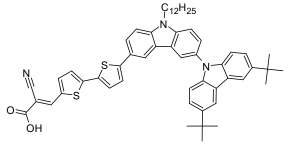 dye43	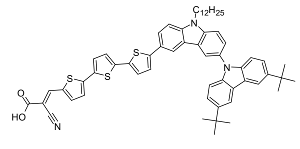 dye44	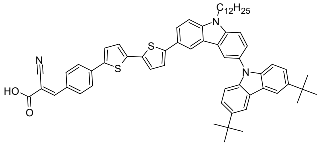 dye45
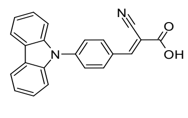 dye46	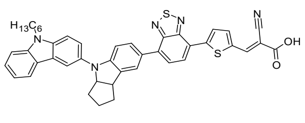 dye47	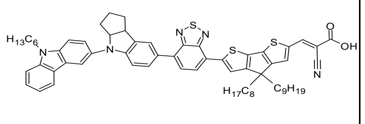 dye48	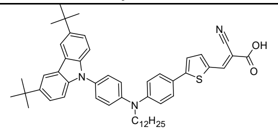 dye49	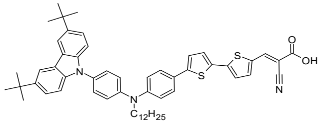 dye50
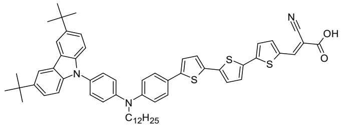 dye51	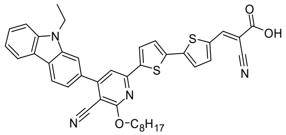 dye52	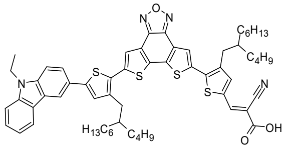 dye53	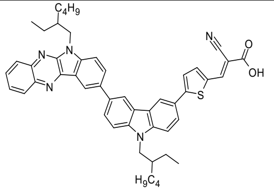 dye54	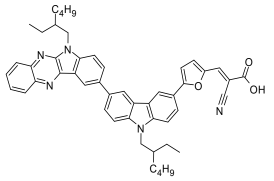 dye55
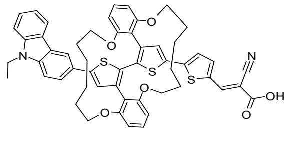 dye56	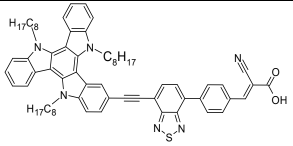 dye57	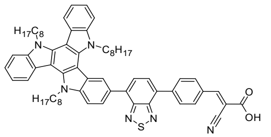 dye58	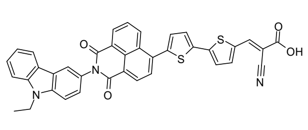 dye59	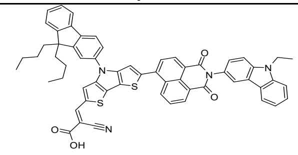 dye60

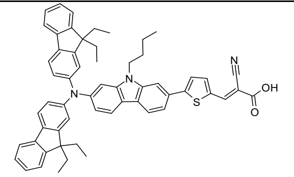 dye61	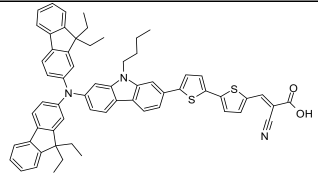 dye62	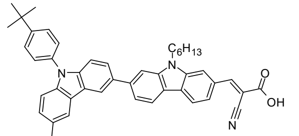 dye63	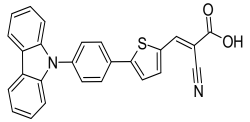 dye64	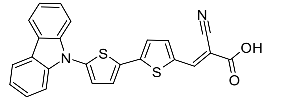 dye65
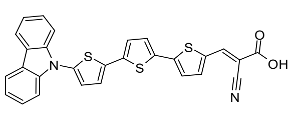 dye66	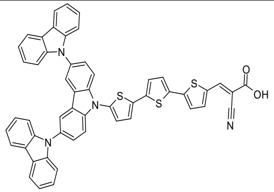 dye67	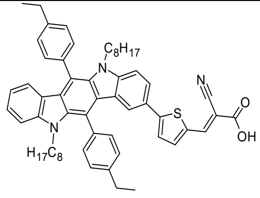 dye68	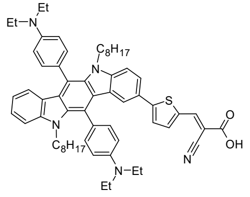 dye69	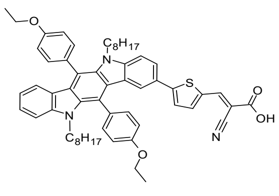 dye70
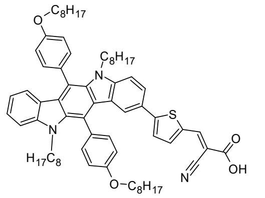 dye71	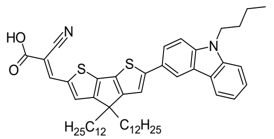 dye72	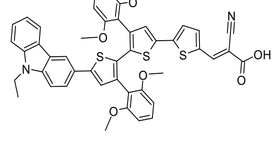 dye73	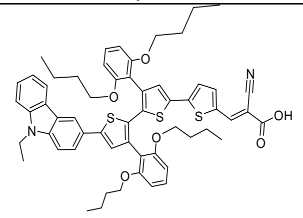 dye74	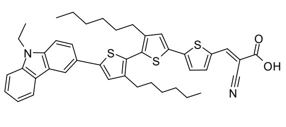 dye75
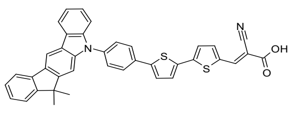 dye76	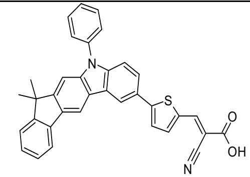 dye77	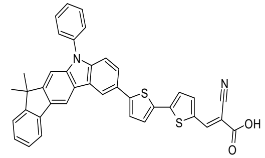 dye78	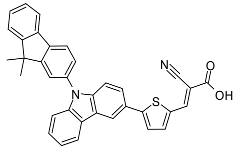 dye79	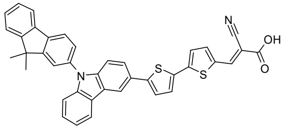 dye80
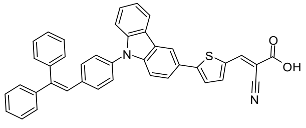 dye81	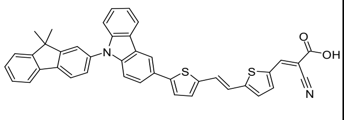 dye82	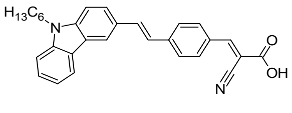 dye83	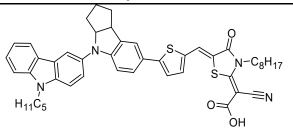 dye84	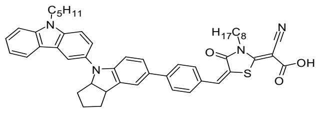 dye85

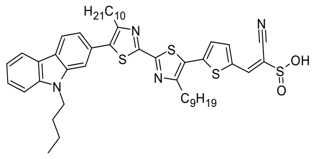<br>dye86	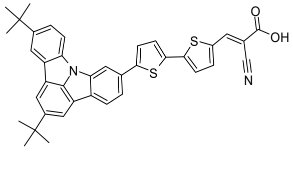<br>dye87	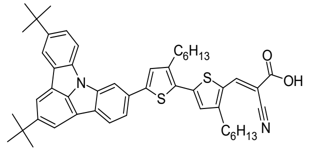<br>dye88	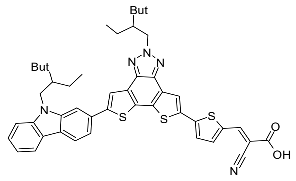<br>dye89	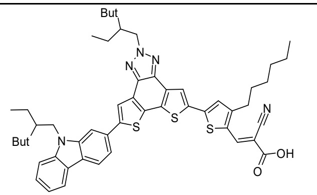<br>dye90
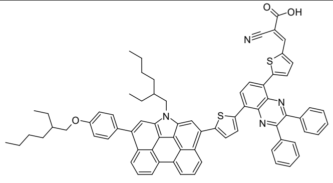<br>dye91	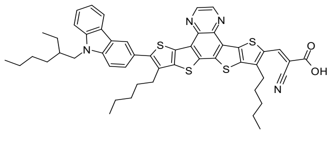<br>dye92	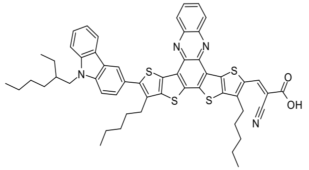<br>dye93	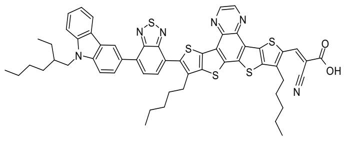<br>dye94	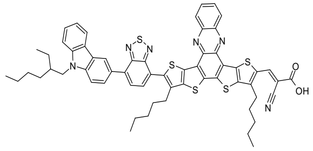<br>dye95
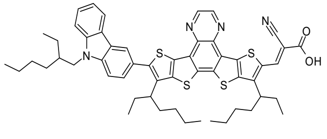<br>dye96	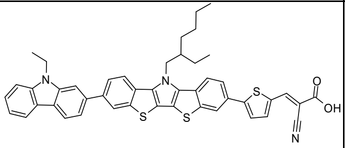<br>dye97	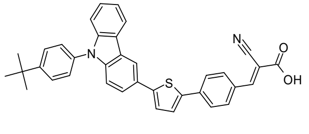<br>dye98	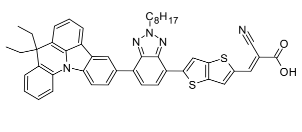<br>dye99	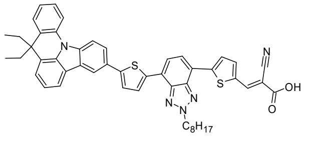<br>dye100
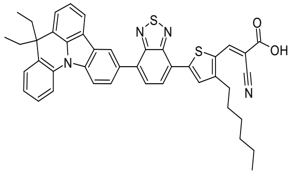<br>dye101	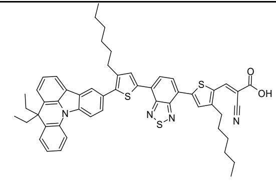<br>dye102	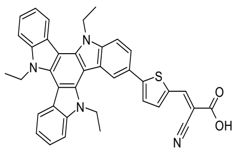<br>dye103	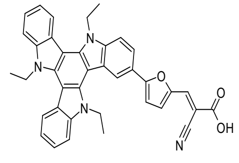<br>dye104	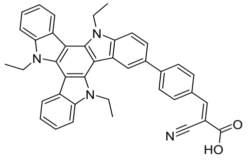<br>dye105
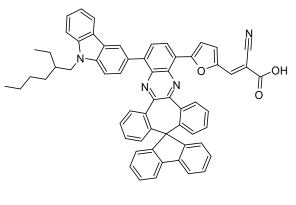<br>dye106	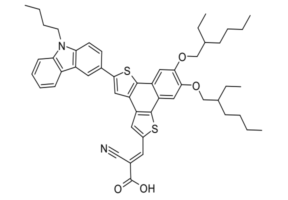<br>dye107	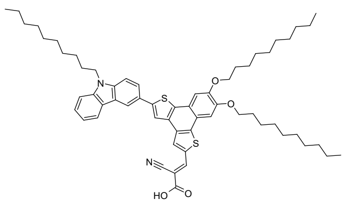<br>dye108	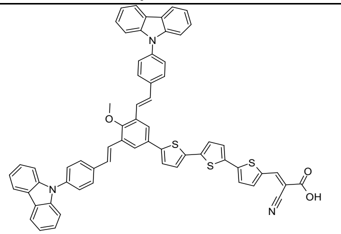<br>dye109	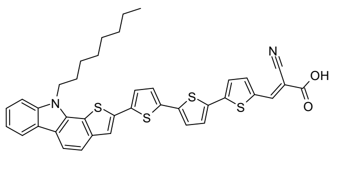<br>dye110

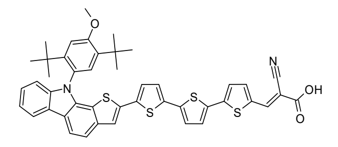 dye111	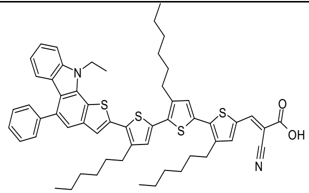 dye112	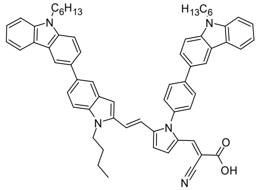 dye113	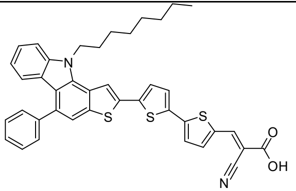 dye114	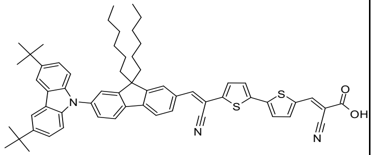 dye115
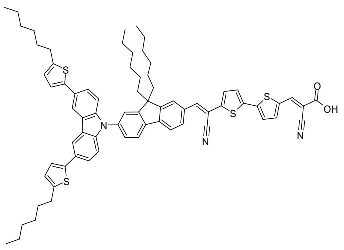 dye116	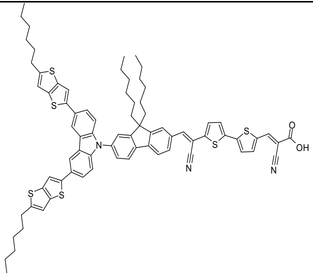 dye117	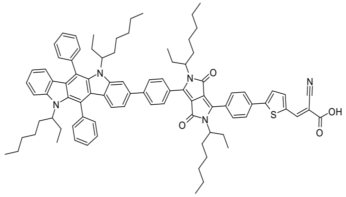 dye118	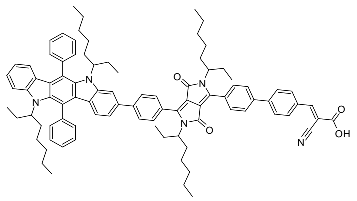 dye119	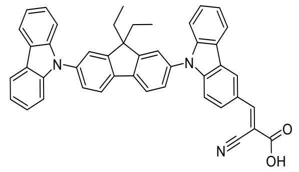 dye120
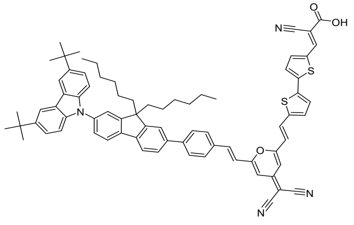 dye121	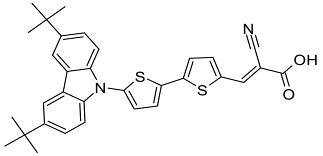 dye122	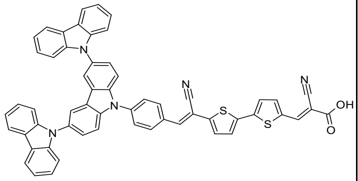 dye123	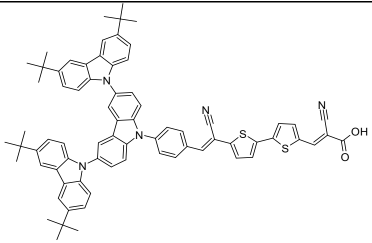 dye124	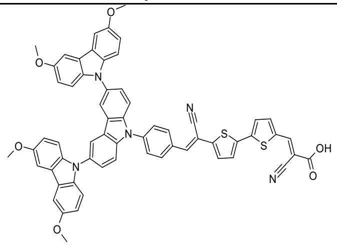 dye125
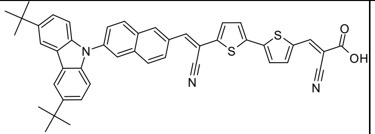 dye126				
